# Crystal structures of {[Cu(Lpn)_2_][Fe(CN)_5_(NO)]·H_2_O}_*n*_ and {[Cu(Lpn)_2_]_3_[Cr(CN)_6_]_2_·5H_2_O}_*n*_ [where Lpn = (*R*)-propane-1,2-di­amine]: two heterometallic chiral cyanide-bridged coordination polymers

**DOI:** 10.1107/S2056989015005253

**Published:** 2015-03-21

**Authors:** Olha Sereda, Helen Stoeckli-Evans

**Affiliations:** a Institute of Physics, University of Neuchâtel, Rue Emile-Argand 11, CH-2000 Neuchâtel, Switzerland

**Keywords:** crystal structure, chiral, bimetallic, cyanide-bridged, (*R*)-propane-1,2-di­amine, coordination polymers, two-dimensional network, three-dimensional framework, hydrogen bonding

## Abstract

The title compounds, *catena*-poly[[[bis­[(*R*)-propane-1,2-di­amine-κ^2^ *N*,*N*′]copper(II)]-μ-cyanido-κ^2^ *N*:*C*-[tris­(cyanido-κ*C*)(nitroso-κ*N*)iron(III)]-μ-cyanido-κ^2^ *C*:*N*] monohydrate], {[Cu(Lpn)_2_][Fe(CN)_5_(NO)]·H_2_O}_*n*_, (I), and poly[[hexa-μ-cyanido-κ^12^ *C*:*N*-hexa­cyanido-κ^6^ *C*-hexa­kis­[(*R*)-propane-1,2-di­amine-κ^2^ *N*,*N*′]dichromium(III)tricopper(II)] penta­hydrate], {[Cu(Lpn)_2_]_3_[Cr(CN)_6_]_2_·5H_2_O}_*n*_, (II) [where Lpn = (*R*)-propane-1,2-di­amine, C_3_H_10_N_2_], are new chiral cyanide-bridged bimetallic coordination polymers. The asymmetric unit of compound (I) is composed of two independent cation–anion units of {[Cu(Lpn)_2_][Fe(CN)_5_)(NO)]} and two water mol­ecules. The Fe^III^ atoms have distorted octa­hedral geometries, while the Cu^II^ atoms can be considered to be penta­coordinate. In the crystal, however, the units align to form zigzag cyanide-bridged chains propagating along [101]. Hence, the Cu^II^ atoms have distorted octa­hedral coordination spheres with extremely long semicoordination Cu—N(cyanido) bridging bonds. The chains are linked by O—H⋯N and N—H⋯N hydrogen bonds, forming two-dimensional networks parallel to (010), and the networks are linked *via* N—H⋯O and N—H⋯N hydrogen bonds, forming a three-dimensional framework. Compound (II) is a two-dimensional cyanide-bridged coordination polymer. The asymmetric unit is composed of two chiral {[Cu(Lpn)_2_][Cr(CN)_6_]}^−^ anions bridged by a chiral [Cu(Lpn)_2_]^2+^ cation and five water mol­ecules of crystallization. Both the Cr^III^ atoms and the central Cu^II^ atom have distorted octa­hedral geometries. The coordination spheres of the outer Cu^II^ atoms of the asymmetric unit can be considered to be penta­coordinate. In the crystal, these units are bridged by long semicoordination Cu—N(cyanide) bridging bonds forming a two-dimensional network, hence these Cu^II^ atoms now have distorted octa­hedral geometries. The networks, which lie parallel to (10-1), are linked *via* O—H⋯O, O—H⋯N, N—H⋯O and N—H⋯N hydrogen bonds involving all five non-coordinating water mol­ecules, the cyanide N atoms and the NH_2_ groups of the Lpn ligands, forming a three-dimensional framework.

## Abstract 2

Two new chiral cyanide-bridged bimetallic coordination polymers involving the ligand (*R*)-propane-1,2-di­amine (Lpn) are described. One compound is a zigzag cyanide-bridged chain polymer in which the asymmetric unit consists of two independent chiral {[Cu(Lpn)_2_][Fe(CN)_5_(NO)]} units and two water mol­ecules, while the second compound is a two-dimensional cyanide-bridged coordination polymer, in which the asymmetric unit consists of two chiral {[Cu(Lpn)_2_][Cr(CN)_6_]}^−^ anions bridged by a chiral [Cu(Lpn)_2_]^2+^ cation and five water mol­ecules.

## Chemical context   

The design of multi-dimensional mol­ecular systems is closely linked to their unique bulk physicochemical properties, such as magnetism (Kahn, 1993 ▸). Examples of these systems include cyanide-bridged complexes, in which a cyanido­metallate anion serves as the bridging moiety in a multi-dimensional structure with a second coordination centre (Fukita *et al.*, 1998 ▸; Ohba *et al.*, 1999 ▸; Tanase & Reedijk, 2006 ▸; Zhang & Luo, 2006 ▸). In this context, heterometallic and chiral frameworks are of particular inter­est (Cui *et al.*, 2002 ▸; Mironov *et al.*, 2004 ▸). A chiral network would allow selective binding of chiral guests, and the presence of different types of metal ions may enable specific tuning of the electronic properties. However, only a few examples of chiral cyanide-bridged bimetallic complexes have been published so far (Coronado *et al.*, 2003 ▸; Imai *et al.*, 2004 ▸; Kaneko *et al.*, 2006 ▸). We report herein on the synthesis and crystal structures of two new chiral cyanide-bridged heterometallic coordination polymers, (I) and (II), synthesized using the chiral ligand (*R*)-propane-1,2-di­amine. Compound (I) is isotypic with [Cu(1,2-pn)_2_][Fe(CN)_5_NO]·H_2_O, synthesized using the racemic form of the same ligand propane-1,2-di­amine (Smékal *et al.*, 2000 ▸).
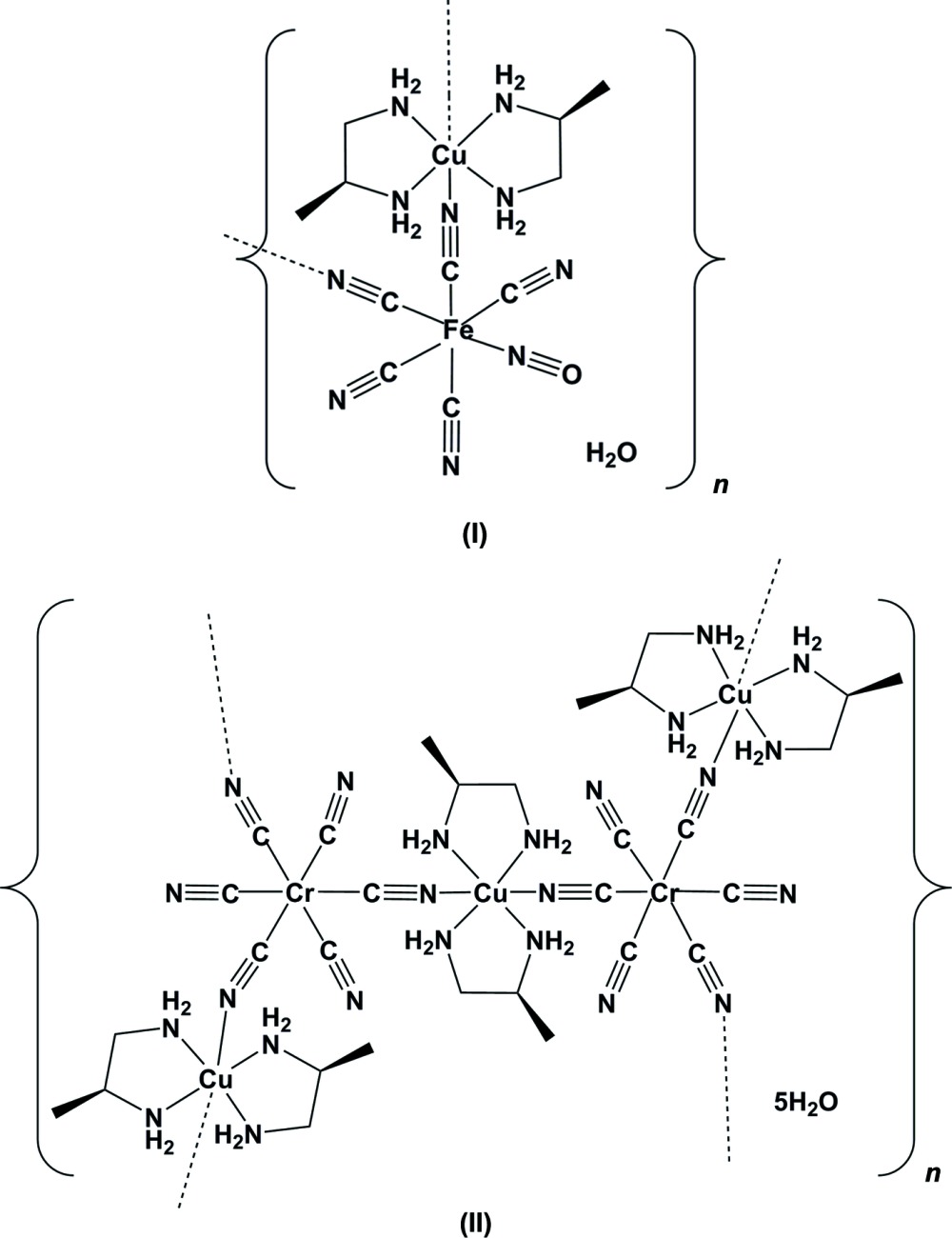


## Structural commentary   

**Figure 1:**
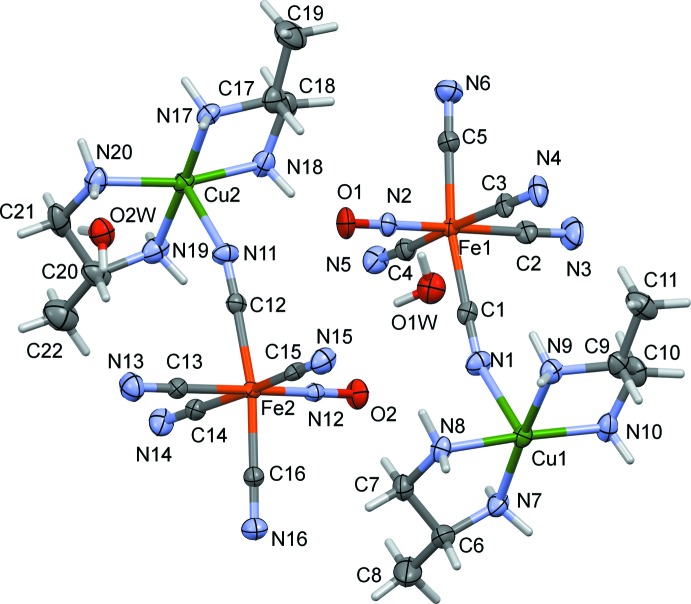
A view of the asymmetric unit of compound (I), showing the atom labelling. Displacement ellipsoids are drawn at the 50% probability level.

The asymmetric unit of complex (I) (Fig. 1 ▸) is composed of two independent cation–anion units of [Cu(Lpn)_2_]^2+^·[Fe(CN)_5_)(NO)]^2−^·H_2_O. Atoms Fe1 and Fe2 have distorted octa­hedral geometries being coordinated by five C atoms from the cyanide ligands (two cyanido groups are bridging and two terminal) and by one N atom, N2 and N12, respectively, from the nitrosyl group. The average Fe—N distance [1.657 (14) Å] is much shorter than the Fe—C distances, which are between 1.926 (5) and 1.954 (6) Å. These values are in good agreement with those reported for other polymeric structures involving nitro­prusside (Shyu *et al.*, 1997 ▸; Chen *et al.*, 1995 ▸). Atoms Cu1 and Cu2 are penta­coordinate. Atom Cu1 has a perfect square-pyramidal geometry with a τ value of 0 (Addison *et al.*, 1984 ▸), while atom Cu2 has a distorted square-pyramidal geometry with a τ value of 0.23. The Cu—N(Lpn) bond lengths vary between 1.998 (5) and 2.026 (5) Å, while the axial bond length Cu1—N1 is 2.333 (5) Å and Cu2—N11 is 2.290 (5) Å.

**Figure 2:**
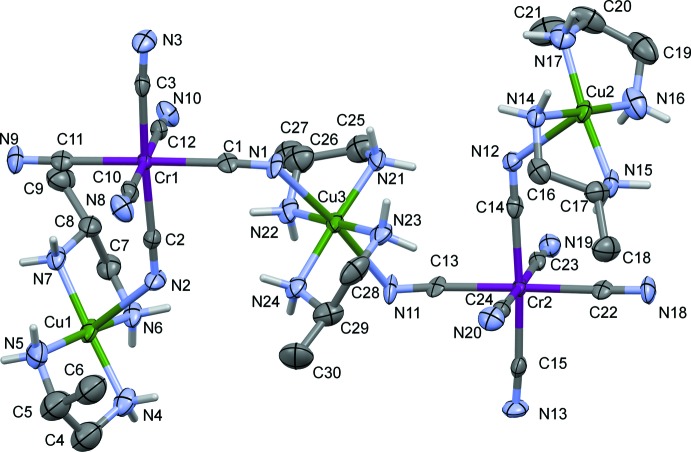
A view of the asymmetric unit of compound (II), showing the atom labelling. Displacement ellipsoids are drawn at the 30% probability level. Water mol­ecules and the C-bound H atoms have been omitted for clarity.

The asymmetric unit of complex (II) (Fig. 2 ▸) consists of two chiral {[Cu(Lpn)_2_][Cr(CN)_6_]}^−^ anions bridged by a chiral [Cu(Lpn)_2_]^2+^ cation. There are also five water mol­ecules of crystallization present. The coordination sphere of the central Cu^II^ atom, Cu3, can be described as elongated octa­hedral, generated by four N atoms of the Lpn ligands and two cyanide N atoms. The outer atoms Cu1 and Cu2 are penta­coordinate; atom Cu1 has a distorted square-pyramidal geometry with a τ value of 0.14 (Addison *et al.*, 1984 ▸), while atom Cu2 has an almost perfect square-pyramidal geometry with a τ value of 0.04. The Cu—N(Lpn) bond lengths vary between 1.960 (12) and 2.020 (10) Å, which is similar to the bond lengths observed in (I) and in a copper(II) complex involving (*S*)-propane-1,2-di­amine (Higashikawa *et al.*, 2007 ▸). The axial bond lengths Cu1—N2 and Cu2—N12 are 2.540 (12) and 2.490 (12) Å, respectively, while those for Cu3 are 2.465 (9) and 2.639 (12) Å for Cu3—N1 and Cu3—N11, respectively. Each Cr^III^ ion has an almost regular octa­hedral coordination geometry. The Cr—C bond lengths are in the range 2.047 (15)–2.081 (15) Å), and the Cr—C≡N bond angles vary over a small range, 174.5 (13)–179.6 (12)°.

## Supra­molecular features   

**Figure 3:**
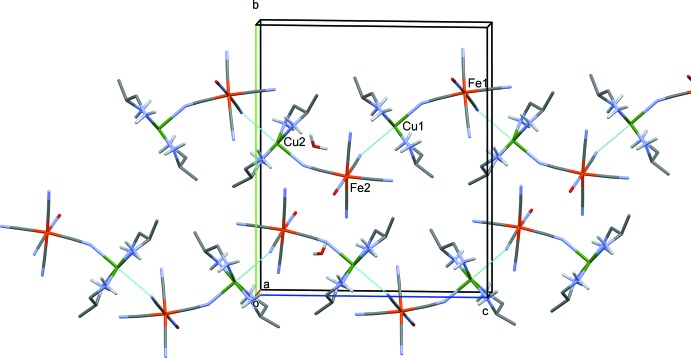
A partial view along the *a* axis of the crystal packing of compound (I), showing the one-dimensional polymer structure (Cu atoms are green, Fe atoms are orange, and bridging Cu—N bonds are thin dashed cyan lines). Water mol­ecules and the C-bound H atoms have been omitted for clarity.

**Figure 4:**
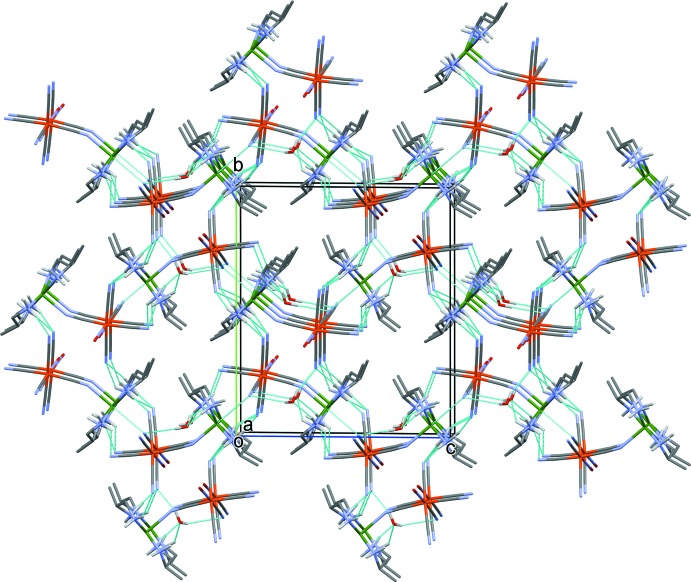
Crystal packing of compound (I), viewed along the *a* axis. Hydrogen bonds are shown as dashed lines (see Table 1 ▸ for details) and C-bound H atoms have been omitted for clarity.

In the crystal of (I), the independent bimetallic units line up to form zigzag polymer chains propagating along [101] (see Fig. 3 ▸). The bridging axial bond lengths are 2.980 (9) and 3.112 (8) Å for Cu1—N13^i^ and Cu2—N3^ii^, respectively [symmetry codes: (i) −*x* + 1, *y* − 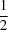, −*z* + 1; (ii) −*x*, *y* + 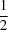, −*z*]. This axial bonding results in distorted octa­hedral coordination spheres for the copper(II) atoms. The extremely long semi-coord­ination Cu—N bonds can be attributed to the co-existence of pseudo-Jahn–Teller elongation and electrostatic inter­actions in the infinite one dimensional chain. A similar geometry has been found in [Cu^II^ *L*_2_][*M* ^II^(CN)_4_]·2H_2_O [*M* ^II^ = Ni^II^, Pt^II^; *L* = *trans*-cyclo­hexane-(1*R*,2*R*)-di­amine] (Akitsu & Einaga, 2006 ▸). Neighbouring chains are linked *via* O—H⋯N and N—H⋯N hydrogen bonds (Table 1 ▸), forming sheets parallel to (010). The sheets are linked *via* N—H⋯O and further N—H⋯N hydrogen bonds, forming a three-dimensional framework (Table 1 ▸ and Fig. 4 ▸).

**Figure 5:**
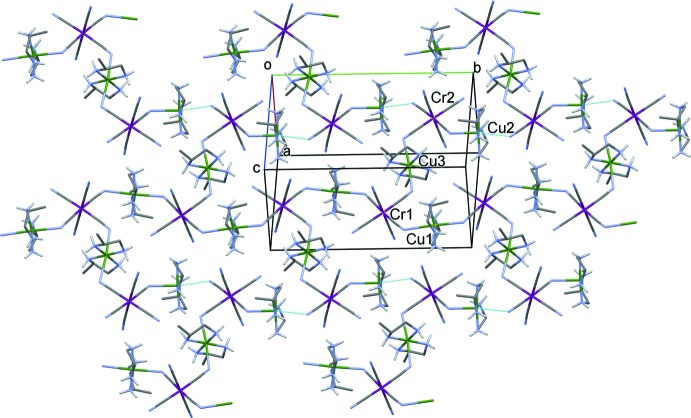
A partial view approximately along [101] of the crystal packing of compound (II), showing the two-dimensional polymer structure (Cu atoms are green, Cr atoms are violet, and bridging Cu—N bonds are thin dashed cyan lines). Water mol­ecules and the C-bound H atoms have been omitted for clarity.

**Figure 6:**
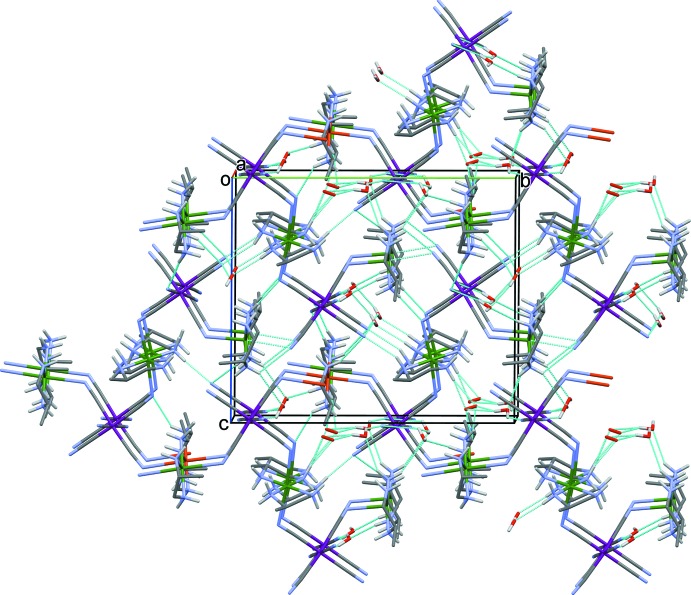
Crystal packing of compound (II), viewed along the *a* axis. Hydrogen bonds are shown as dashed lines (see Table 2 ▸ for details) and C-bound H atoms have been omitted for clarity.

In the crystal of (II), the cation-anion units are linked to form two-dimensional networks lying parallel to (10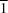) (see Fig. 5 ▸). The bridging Cu—N(cyanido) bond lengths, Cu1—N3^iii^ and Cu2—N13^iv^, are 2.698 (14) and 2.860 (14) Å, respectively [symmetry codes: (iii) −*x* + 1, *y* − 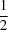, −*z*; (iv) −*x* + 2, *y* + 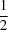, −*z* + 1]. Thus, as for complex (I), atoms Cu1 and Cu2 have octa­hedral coordination spheres with a strong pseudo-Jahn–Teller effect. Closely related two-dimensional bimetallic systems have been found in iron(III) analogues, where [Fe(CN)_6_]^3−^ anions binds to three adjacent nickel atoms (Kou *et al.*, 1999 ▸, 2000 ▸). The two-dimensional networks of (II) (Fig. 5 ▸) are linked by a series of O—H⋯O, O—H⋯N, N—H⋯O and N—H⋯N hydrogen bonds, involving the water mol­ecules, the cyanide N atoms and the NH_2_ groups of the Lpn ligands, forming a three-dimensional framework (Fig. 6 ▸ and Table 2 ▸).

## Database survey   

A search of the Cambridge Structural Database (Version 5.36, last update November 2014; Groom & Allen, 2014 ▸) gave 49 hits for bimetallic cyanide-bridged complexes involving trans­ition metals and the ligand propane-1,2-di­amine. Of these, only two complexes involved (*R*)-propane-1,2-di­amine, *viz. catena*-[tris­(μ_2_-cyanido)­cyanido­[(*R*)-1,2-di­amino­propane]copper(II)nickel(II) hemihydrate clathrate] (IZEPOS; Imai *et al.*, 2003 ▸) and *catena*-[hepta­deca­kis­(μ_2_-cyanido-κ^2^ *C*:*N*)tetraaqua­penta­deca­cyanido­hexa­kis­[(*R*)-propane-1,2-di­amine-κ^2^ *N*,*N*′]hexa­copper(II)tetra­tungsten(V) hydrate] (YIMBEC; Higashikawa *et al.*, 2007 ▸). Two complexes involved (*S*)-propane-1,2-di­amine, *viz. catena*-[potassium (*S*)-1-amino-2-ammonio­propane tetra­kis­(μ_2_-cyanido)­dicyanido­[(*S*)-1,2-diamino­propane-κ^2^ *N*,*N*′]chromiummanganese(II) (*S*)-1,2-diamino­propane] (IDEBOI; Inoue *et al.*, 2001 ▸) and *catena*-[hepta­deca­kis­(μ_2_-cyanido-κ^2^ *C*:*N*)tetra­aqua­penta­decacyanidohexa­kis­[(*S*)-propane-1,2-di­amine-κ^2^ *N*,*N*′]hexacopper(II)tetra­tungsten(V) hydrate] (YIMBAY; Higashikawa *et al.*, 2007 ▸). They were studied principally for their magnetic properties, compound IDEBOI being a ferrimagnet, while the other three compounds have one- or two-dimensional anti­ferromagnetic properties.

## Synthesis and crystallization   

**Compound (I)**: (*R*)-propane-1,2-di­amine (Lpn) was synthesized according to a reported procedure (Bernauer, 1971 ▸). The pH of an aqueous solution of Lpn·HCl (0.1 mmol in 1 ml of water) was adjusted to 7–8 by the addition of an aqueous solution of KOH (0.12 mmol in 0.3 ml of water). To this mixture, a solution of CuSO_4_·5H_2_O (0.1 mmol) in 0.8 ml of water was added under an argon atmosphere. A glass tube (*ca* 8 mm diameter, *ca* 20 cm long) was charged with this solution, and a mixture of methanol and H_2_O (1:2, 1.5 ml) was gently added as a buffer layer. A solution of Na_2_[Fe(CN)_5_NO] (0.07 mmol) in methanol/H_2_O (1:1, 1 ml) was then added carefully as a third layer under an argon atmosphere, and then the tube was sealed. Crystals of complex (I) grew as violet blocks after several weeks. Elemental analysis for C_11_H_22_N_10_CuFeO_2_, found: C, 29.86; H, 5.07; N, 31.93%. calc: C, 29.64; H, 4.97 N, 31.42%.

**Compound (II)**: Dark-blue block-like crystals of compound (II) were prepared in a similar manner to those of (I), but this time using K_3_[Cr(CN)_6_] instead of Na_2_[Fe(CN)_5_NO]. Elemental analysis for C_30_H_70_N_24_Cu_3_Cr_2_O_5_, found: C, 30.87; H, 5.80; N, 28.41%. calc: C, 31.56; H, 6.18 N, 29.44%.

## Refinement details   

Crystal data, data collection and structure refinement details are summarized in Table 3 ▸. For both compounds, the water mol­ecule H atoms were located in difference Fourier maps and refined with distance restraints of O—H = 0.84 (2) Å and with *U*_iso_(H) = 1.5*U*_eq_(O). The N- and C-bound H atoms were included in calculated positions and treated as riding atoms: N—H = 0.89 Å, C—H = 0.98–1.00 Å with *U*_iso_(H) = 1.5*U*_eq_(C) for methyl H atoms and 1.2*U*_eq_(N,C) for other H atoms. It was not possible to locate the H atoms of the disordered water mol­ecule, OW5*A*/OW5*B*, in compound (II).

## Supplementary Material

Crystal structure: contains datablock(s) I, II, Global. DOI: 10.1107/S2056989015005253/hb7374sup1.cif

Structure factors: contains datablock(s) I. DOI: 10.1107/S2056989015005253/hb7374Isup2.hkl

Structure factors: contains datablock(s) II. DOI: 10.1107/S2056989015005253/hb7374IIsup3.hkl

CCDC references: 691330, 691331

Additional supporting information:  crystallographic information; 3D view; checkCIF report

## Figures and Tables

**Table 1:** Hydrogen-bond geometry (Å, °) for (I)

*D*—H⋯*A*	*D*—H	H⋯*A*	*D*⋯*A*	*D*—H⋯*A*
N7—H7*X*⋯N15^i^	0.89	2.39	3.257 (8)	166
N7—H7*Y*⋯N14^ii^	0.89	2.12	3.008 (8)	173
N8—H8*X*⋯N14^iii^	0.89	2.27	3.120 (8)	160
N8—H8*Y*⋯N15	0.89	2.45	3.209 (7)	144
N9—H9*X*⋯O1*W*	0.89	2.52	3.207 (7)	135
N9—H9*Y*⋯N16^iii^	0.89	2.39	3.157 (7)	144
N10—H10*X*⋯N16^ii^	0.89	2.52	3.189 (7)	132
N10—H10*Y*⋯O1*W* ^i^	0.89	2.11	2.962 (7)	159
N17—H17*X*⋯N4^iv^	0.89	2.22	3.051 (8)	155
N17—H17*Y*⋯N5^v^	0.89	2.32	3.197 (7)	169
N18—H18*X*⋯N5	0.89	2.37	3.224 (8)	161
N18—H18*Y*⋯N4^vi^	0.89	2.27	3.080 (8)	151
N19—H19*X*⋯N6^vi^	0.89	2.44	3.295 (8)	160
N19—H19*Y*⋯O2*W* ^i^	0.89	2.30	3.142 (8)	159
N20—H20*X*⋯O2*W*	0.89	2.11	2.990 (8)	172
O1*W*—H1*WB*⋯N15	0.84 (3)	2.11 (3)	2.928 (7)	163 (6)
O2*W*—H2*WA*⋯N13	0.83 (3)	2.65 (4)	3.419 (9)	155 (6)

Symmetry codes: (i) 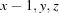; (ii) 
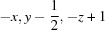
; (iii) 
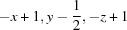
; (iv) 
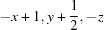
; (v) 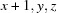; (vi) 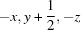.

**Table 2:** Hydrogen-bond geometry (Å, °) for (II)

*D*—H⋯*A*	*D*—H	H⋯*A*	*D*⋯*A*	*D*—H⋯*A*
N4—H4*X*⋯N19^i^	0.89	2.27	3.153 (16)	171
N5—H5*X*⋯O5*WA* ^ii^	0.89	2.24	3.04 (4)	150
N5—H5*X*⋯O5*WB* ^ii^	0.89	2.22	2.89 (3)	133
N5—H5*Y*⋯O4*W*	0.89	2.50	3.177 (17)	133
N6—H6*X*⋯N18^i^	0.89	2.56	3.373 (17)	152
N7—H7*X*⋯N1^ii^	0.89	2.49	3.219 (15)	139
N14—H14*Y*⋯N9^iii^	0.89	2.62	3.360 (16)	142
N15—H15*Y*⋯N11^iv^	0.89	2.27	3.156 (15)	177
N16—H16*Y*⋯O1*W* ^v^	0.89	2.32	3.159 (16)	158
N17—H17*X*⋯O2*W*	0.89	2.33	3.132 (18)	150
N17—H17*Y*⋯N13^iv^	0.89	2.69	3.166 (19)	115
N17—H17*Y*⋯O4*W* ^vi^	0.89	2.60	3.361 (18)	145
N21—H21*X*⋯O5*WA*	0.89	2.16	3.04 (5)	170
N21—H21*X*⋯O5*WB*	0.89	2.02	2.88 (3)	163
N21—H21*Y*⋯N12	0.89	2.51	3.376 (17)	164
N22—H22*X*⋯N18^i^	0.89	2.43	3.262 (17)	155
N23—H23*X*⋯N20	0.89	2.68	3.445 (18)	144
N23—H23*Y*⋯N9^iii^	0.89	2.20	3.086 (17)	172
N24—H24*Y*⋯O3*W*	0.89	2.09	2.969 (18)	167
O1*W*—H1*WA*⋯N19^vii^	0.85 (3)	2.14 (5)	2.972 (16)	167 (16)
O1*W*—H1*WB*⋯N20	0.84 (3)	2.00 (5)	2.822 (15)	164 (14)
O2*W*—H2*WA*⋯N10^viii^	0.85 (3)	2.09 (10)	2.811 (17)	143 (15)
O2*W*—H2*WB*⋯O5*WA*	0.85 (3)	1.78 (9)	2.56 (6)	153 (16)
O2*W*—H2*WB*⋯O5*WB*	0.85 (3)	2.01 (8)	2.85 (7)	169 (20)
O3*W*—H3*WA*⋯N18^i^	0.85 (3)	2.27 (14)	2.982 (19)	142 (20)
O3*W*—H3*WB*⋯O1*W* ^ix^	0.85 (3)	1.92 (10)	2.712 (17)	156 (21)
O4*W*—H4*WA*⋯N10^ii^	0.84 (3)	2.53 (18)	3.104 (17)	126 (18)
O4*W*—H4*WB*⋯N8^x^	0.84 (3)	2.16 (13)	2.883 (16)	145 (19)

Symmetry codes: (i) 
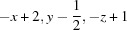
; (ii) 
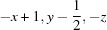
; (iii) 
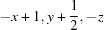
; (iv) 
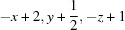
; (v) 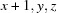; (vi) 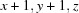; (vii) 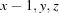; (viii) 
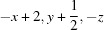
; (ix) 
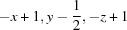
; (x) 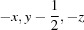.

**Table 3:** Experimental details

	(I)	(II)
Crystal data		
Chemical formula	[CuFe(C_3_H_10_N_2_)_2_(CN)_5_(NO)]·H_2_O	[Cr_2_Cu_3_(CN)_12_(C_3_H_10_N_2_)_6_]·5H_2_O
*M*_r_	445.77	1141.72
Crystal system, space group	Monoclinic, *P*2_1_	Monoclinic, *P*2_1_
Temperature (K)	173	173
*a*, *b*, *c* (Å)	6.7987 (3), 17.891 (1), 15.7161 (8)	10.1474 (10), 17.6136 (10), 15.5376 (14)
β (°)	100.482 (4)	103.973 (11)
*V* (Å^3^)	1879.73 (17)	2694.9 (4)
*Z*	4	2
Radiation type	Mo *K*α	Mo *K*α
μ (mm^−1^)	1.93	1.61
Crystal size (mm)	0.45 × 0.38 × 0.35	0.40 × 0.30 × 0.30

Data collection		
Diffractometer	Stoe IPDS 2	Stoe IPDS 2
Absorption correction	Multi-scan (*MULABS* in *PLATON*; Spek, 2009 ▸)	Multi-scan (*MULABS* in *PLATON*; Spek, 2009 ▸)
*T*_min_, *T*_max_	0.572, 0.740	0.583, 0.678
No. of measured, independent and observed [*I* > 2σ(*I*)] reflections	22802, 9961, 8537	21461, 10308, 4948
*R*_int_	0.033	0.085
(sin θ/λ)_max_ (Å^−1^)	0.688	0.620

Refinement		
*R*[*F* ^2^ > 2σ(*F* ^2^)], *wR*(*F* ^2^), *S*	0.031, 0.078, 1.03	0.052, 0.131, 0.79
No. of reflections	9961	10308
No. of parameters	467	594
No. of restraints	7	14
H-atom treatment	H atoms treated by a mixture of independent and constrained refinement	H atoms treated by a mixture of independent and constrained refinement
Δρ_max_, Δρ_min_ (e Å^−3^)	0.55, −0.44	0.61, −1.05
Absolute structure	Flack *x* determined using 3691 quotients [(*I* ^+^)−(*I* ^−^)]/[(*I* ^+^)+(*I* ^−^)] (Parsons *et al.*, 2013 ▸).	Flack *x* determined using 1754 quotients [(*I* ^+^)−(*I* ^−^)]/[(*I* ^+^)+(*I* ^−^)] (Parsons *et al.*, 2013 ▸)
Absolute structure parameter	0.038 (15)	0.00 (3)

Computer programs: *X-AREA* and *X-RED32* (Stoe & Cie, 2009 ▸), *SHELXS97* (Sheldrick, 2008 ▸), *SHELXL2014* (Sheldrick, 2015 ▸), *PLATON* (Spek, 2009 ▸), *Mercury* (Macrae *et al.*, 2008 ▸) and *publCIF* (Westrip, 2010 ▸).

## supplementary crystallographic information

### (I) catena-Poly[[[bis[(R)-propane-1,2-diamine-κ2N,N']copper(II)]-µ-cyanido-κ2N:C-[tris(cyanido-κC)(nitroso-κN)iron(III)]-µ-cyanido-κ2C:N] monohydrate] Crystal data

**Table:**

[CuFe(C_3_H_10_N_2_)_2_(CN)_5_(NO)]·H_2_O	*F*(000) = 916
*M**_r_* = 445.77	*D*_x_ = 1.575 Mg m^−^^3^
Monoclinic, *P*2_1_	Mo *K*α radiation, λ = 0.71073 Å
*a* = 6.7987 (3) Å	Cell parameters from 22904 reflections
*b* = 17.891 (1) Å	θ = 1.8–29.3°
*c* = 15.7161 (8) Å	µ = 1.93 mm^−^^1^
β = 100.482 (4)°	*T* = 173 K
*V* = 1879.73 (17) Å^3^	Plate, violet
*Z* = 4	0.45 × 0.38 × 0.35 mm

### (I) catena-Poly[[[bis[(R)-propane-1,2-diamine-κ2N,N']copper(II)]-µ-cyanido-κ2N:C-[tris(cyanido-κC)(nitroso-κN)iron(III)]-µ-cyanido-κ2C:N] monohydrate] Data collection

**Table:**

Stoe IPDS 2 diffractometer	9961 independent reflections
Radiation source: fine-focus sealed tube	8537 reflections with *I* > 2σ(*I*)
Plane graphite monochromator	*R*_int_ = 0.033
φ + ω scans	θ_max_ = 29.3°, θ_min_ = 1.7°
Absorption correction: multi-scan (MULABS in PLATON; Spek, 2009)	*h* = −8→9
*T*_min_ = 0.572, *T*_max_ = 0.740	*k* = −24→24
22802 measured reflections	*l* = −21→21

### (I) catena-Poly[[[bis[(R)-propane-1,2-diamine-κ2N,N']copper(II)]-µ-cyanido-κ2N:C-[tris(cyanido-κC)(nitroso-κN)iron(III)]-µ-cyanido-κ2C:N] monohydrate] Refinement

**Table:**

Refinement on *F*^2^	Secondary atom site location: difference Fourier map
Least-squares matrix: full	Hydrogen site location: mixed
*R*[*F*^2^ > 2σ(*F*^2^)] = 0.031	H atoms treated by a mixture of independent and constrained refinement
*wR*(*F*^2^) = 0.078	*w* = 1/[σ^2^(*F*_o_^2^) + (0.0453*P*)^2^ + 0.0779*P*] where *P* = (*F*_o_^2^ + 2*F*_c_^2^)/3
*S* = 1.03	(Δ/σ)_max_ < 0.001
9961 reflections	Δρ_max_ = 0.55 e Å^−^^3^
467 parameters	Δρ_min_ = −0.44 e Å^−^^3^
7 restraints	Absolute structure: Flack *x* determined using 3691 quotients [(*I*^+^)-(*I*^-^)]/[(*I*^+^)+(*I*^-^)] (Parsons *et al.*, 2013).
Primary atom site location: structure-invariant direct methods	Absolute structure parameter: 0.038 (15)

### (I) catena-Poly[[[bis[(R)-propane-1,2-diamine-κ2N,N']copper(II)]-µ-cyanido-κ2N:C-[tris(cyanido-κC)(nitroso-κN)iron(III)]-µ-cyanido-κ2C:N] monohydrate] Special details

**Table:**

Geometry. All e.s.d.'s (except the e.s.d. in the dihedral angle between two l.s. planes) are estimated using the full covariance matrix. The cell e.s.d.'s are taken into account individually in the estimation of e.s.d.'s in distances, angles and torsion angles; correlations between e.s.d.'s in cell parameters are only used when they are defined by crystal symmetry. An approximate (isotropic) treatment of cell e.s.d.'s is used for estimating e.s.d.'s involving l.s. planes.

### (I) catena-Poly[[[bis[(R)-propane-1,2-diamine-κ2N,N']copper(II)]-µ-cyanido-κ2N:C-[tris(cyanido-κC)(nitroso-κN)iron(III)]-µ-cyanido-κ2C:N] monohydrate] Fractional atomic coordinates and isotropic or equivalent isotropic displacement parameters (Å^2^)

**Table:**

	*x*	*y*	*z*	*U*_iso_*/*U*_eq_	
Cu1	0.10706 (10)	0.11302 (3)	0.41868 (5)	0.02243 (16)	
Fe1	0.07542 (12)	0.24094 (3)	0.11676 (5)	0.01767 (16)	
O1	0.4628 (7)	0.2972 (2)	0.1620 (3)	0.0346 (11)	
N1	0.0460 (9)	0.1979 (3)	0.3043 (4)	0.0298 (12)	
N2	0.3065 (8)	0.2752 (2)	0.1432 (3)	0.0231 (10)	
N3	−0.3427 (9)	0.1681 (4)	0.0694 (5)	0.0452 (16)	
N4	0.2137 (9)	0.0800 (3)	0.0924 (5)	0.0362 (14)	
N5	−0.1151 (9)	0.3962 (3)	0.1278 (4)	0.0329 (12)	
N6	0.0371 (9)	0.2626 (3)	−0.0800 (4)	0.0387 (12)	
N7	−0.0285 (8)	0.1791 (3)	0.4932 (4)	0.0295 (11)	
H7X	−0.1059	0.2120	0.4603	0.035*	
H7Y	−0.1057	0.1516	0.5210	0.035*	
N8	0.3547 (7)	0.1695 (2)	0.4694 (4)	0.0257 (10)	
H8X	0.4323	0.1415	0.5087	0.031*	
H8Y	0.4234	0.1817	0.4283	0.031*	
N9	0.2476 (8)	0.0400 (3)	0.3514 (3)	0.0279 (10)	
H9X	0.2753	0.0621	0.3042	0.033*	
H9Y	0.3622	0.0253	0.3839	0.033*	
N10	−0.1344 (8)	0.0507 (3)	0.3756 (4)	0.0317 (11)	
H10X	−0.1620	0.0221	0.4183	0.038*	
H10Y	−0.2395	0.0800	0.3574	0.038*	
C1	0.0529 (8)	0.2162 (3)	0.2339 (4)	0.0215 (11)	
C2	−0.1916 (9)	0.1970 (3)	0.0875 (4)	0.0253 (12)	
C3	0.1650 (8)	0.1403 (3)	0.1026 (4)	0.0233 (12)	
C4	−0.0513 (9)	0.3374 (3)	0.1251 (4)	0.0246 (12)	
C5	0.0524 (9)	0.2554 (3)	−0.0071 (4)	0.0278 (12)	
C6	0.1233 (9)	0.2186 (3)	0.5568 (3)	0.0363 (11)	
H6	0.1732	0.1842	0.6061	0.044*	
C7	0.2936 (7)	0.2377 (2)	0.5113 (3)	0.0310 (9)	
H7A	0.2510	0.2768	0.4672	0.037*	
H7B	0.4079	0.2573	0.5536	0.037*	
C8	0.0405 (14)	0.2889 (4)	0.5915 (6)	0.058 (2)	
H8A	−0.0820	0.2766	0.6131	0.087*	
H8B	0.0102	0.3259	0.5450	0.087*	
H8C	0.1398	0.3094	0.6388	0.087*	
C9	0.1164 (7)	−0.0257 (2)	0.3268 (3)	0.0310 (9)	
H9	0.1262	−0.0588	0.3786	0.037*	
C10	−0.0953 (7)	0.0037 (3)	0.3051 (4)	0.0341 (10)	
H10A	−0.1128	0.0330	0.2508	0.041*	
H10B	−0.1908	−0.0386	0.2964	0.041*	
C11	0.1796 (12)	−0.0705 (4)	0.2547 (5)	0.0423 (15)	
H11A	0.3156	−0.0895	0.2739	0.063*	
H11B	0.1765	−0.0384	0.2039	0.063*	
H11C	0.0876	−0.1126	0.2396	0.063*	
Cu2	0.39302 (11)	0.55505 (3)	0.08355 (5)	0.02290 (16)	
Fe2	0.41932 (12)	0.43450 (3)	0.38348 (5)	0.01729 (16)	
O2	0.0342 (6)	0.3768 (2)	0.3377 (3)	0.0317 (10)	
N11	0.4582 (9)	0.4728 (3)	0.1969 (4)	0.0273 (11)	
N12	0.1918 (7)	0.4003 (2)	0.3562 (3)	0.0191 (9)	
N13	0.8371 (9)	0.5054 (3)	0.4342 (4)	0.0370 (13)	
N14	0.2910 (8)	0.5974 (3)	0.4029 (4)	0.0330 (12)	
N15	0.6236 (9)	0.2807 (3)	0.3783 (4)	0.0333 (13)	
N16	0.4194 (8)	0.4211 (3)	0.5782 (4)	0.0325 (11)	
N17	0.5330 (7)	0.4883 (3)	0.0108 (3)	0.0251 (10)	
H17X	0.5716	0.5147	−0.0313	0.030*	
H17Y	0.6413	0.4685	0.0433	0.030*	
N18	0.1419 (8)	0.5031 (3)	0.0242 (4)	0.0304 (12)	
H18X	0.0998	0.4716	0.0609	0.036*	
H18Y	0.0454	0.5363	0.0069	0.036*	
N19	0.2644 (9)	0.6247 (3)	0.1572 (4)	0.0346 (12)	
H19X	0.1798	0.6553	0.1241	0.041*	
H19Y	0.1969	0.5989	0.1908	0.041*	
N20	0.6299 (9)	0.6241 (3)	0.1114 (4)	0.0320 (11)	
H20X	0.7298	0.6010	0.1460	0.038*	
H20Y	0.6719	0.6371	0.0631	0.038*	
C12	0.4471 (9)	0.4562 (3)	0.2656 (4)	0.0222 (12)	
C13	0.6835 (10)	0.4773 (3)	0.4159 (4)	0.0252 (12)	
C14	0.3364 (9)	0.5368 (3)	0.3945 (4)	0.0242 (12)	
C15	0.5529 (9)	0.3388 (3)	0.3807 (4)	0.0205 (11)	
C16	0.4259 (8)	0.4240 (3)	0.5064 (4)	0.0231 (11)	
C17	0.3943 (8)	0.4284 (2)	−0.0270 (4)	0.0328 (10)	
H17	0.3918	0.3890	0.0180	0.039*	
C18	0.1901 (8)	0.4623 (3)	−0.0496 (3)	0.0361 (10)	
H18A	0.1852	0.4967	−0.0992	0.043*	
H18B	0.0898	0.4224	−0.0668	0.043*	
C19	0.4588 (15)	0.3922 (4)	−0.1060 (6)	0.055 (2)	
H19A	0.4664	0.4306	−0.1498	0.082*	
H19B	0.3608	0.3541	−0.1302	0.082*	
H19C	0.5903	0.3689	−0.0885	0.082*	
C20	0.4301 (9)	0.6681 (3)	0.2113 (3)	0.0406 (11)	
H20	0.5033	0.6338	0.2565	0.049*	
C21	0.5683 (9)	0.6910 (3)	0.1547 (4)	0.0433 (13)	
H21A	0.6871	0.7156	0.1892	0.052*	
H21B	0.5018	0.7271	0.1110	0.052*	
C22	0.3594 (13)	0.7349 (4)	0.2563 (6)	0.053 (2)	
H22A	0.2714	0.7181	0.2955	0.080*	
H22B	0.2858	0.7690	0.2131	0.080*	
H22C	0.4751	0.7609	0.2897	0.080*	
O1W	0.5517 (7)	0.1452 (3)	0.2726 (4)	0.0378 (11)	
H1WA	0.630 (9)	0.142 (3)	0.239 (4)	0.057*	
H1WB	0.568 (10)	0.189 (2)	0.293 (4)	0.057*	
O2W	0.9350 (8)	0.5353 (2)	0.2311 (3)	0.0369 (11)	
H2WA	0.953 (10)	0.526 (3)	0.284 (2)	0.055*	
H2WB	0.890 (10)	0.576 (2)	0.219 (4)	0.055*	

### (I) catena-Poly[[[bis[(R)-propane-1,2-diamine-κ2N,N']copper(II)]-µ-cyanido-κ2N:C-[tris(cyanido-κC)(nitroso-κN)iron(III)]-µ-cyanido-κ2C:N] monohydrate] Atomic displacement parameters (Å^2^)

**Table:**

	*U*^11^	*U*^22^	*U*^33^	*U*^12^	*U*^13^	*U*^23^
Cu1	0.0191 (3)	0.0216 (3)	0.0265 (4)	0.0003 (2)	0.0040 (3)	0.0021 (3)
Fe1	0.0175 (4)	0.0162 (3)	0.0195 (4)	−0.0007 (3)	0.0038 (3)	−0.0003 (3)
O1	0.029 (2)	0.0278 (19)	0.046 (3)	−0.0116 (17)	0.002 (2)	−0.0036 (18)
N1	0.034 (3)	0.026 (2)	0.031 (3)	0.005 (2)	0.008 (2)	0.006 (2)
N2	0.028 (3)	0.0141 (17)	0.027 (3)	−0.0008 (17)	0.005 (2)	−0.0002 (17)
N3	0.028 (3)	0.050 (3)	0.059 (4)	−0.015 (3)	0.012 (3)	−0.011 (3)
N4	0.032 (3)	0.023 (2)	0.056 (4)	−0.0056 (19)	0.013 (3)	−0.013 (2)
N5	0.032 (3)	0.027 (2)	0.042 (3)	0.0061 (19)	0.012 (2)	0.003 (2)
N6	0.041 (3)	0.049 (3)	0.026 (2)	−0.007 (2)	0.0034 (19)	−0.0001 (19)
N7	0.031 (3)	0.026 (2)	0.035 (3)	−0.0009 (19)	0.015 (2)	0.002 (2)
N8	0.021 (2)	0.023 (2)	0.033 (3)	0.0003 (17)	0.005 (2)	0.0030 (18)
N9	0.032 (2)	0.0208 (18)	0.032 (2)	0.0047 (16)	0.0090 (19)	0.0027 (16)
N10	0.027 (2)	0.028 (2)	0.037 (3)	−0.0066 (18)	−0.001 (2)	0.0047 (19)
C1	0.018 (3)	0.018 (2)	0.029 (3)	0.0020 (18)	0.005 (2)	0.002 (2)
C2	0.019 (3)	0.027 (2)	0.031 (3)	0.0014 (19)	0.008 (2)	−0.004 (2)
C3	0.014 (2)	0.022 (2)	0.035 (3)	−0.0017 (18)	0.010 (2)	−0.007 (2)
C4	0.027 (3)	0.021 (2)	0.025 (3)	−0.001 (2)	0.004 (2)	−0.0014 (19)
C5	0.032 (3)	0.025 (2)	0.026 (3)	−0.0048 (19)	0.006 (2)	−0.0023 (19)
C6	0.052 (3)	0.031 (2)	0.029 (2)	−0.009 (2)	0.013 (2)	−0.0021 (18)
C7	0.035 (2)	0.0229 (18)	0.036 (2)	−0.0059 (16)	0.0075 (19)	−0.0023 (17)
C8	0.072 (5)	0.048 (4)	0.067 (5)	−0.018 (3)	0.044 (4)	−0.021 (3)
C9	0.041 (2)	0.0195 (17)	0.030 (2)	0.0031 (16)	−0.0007 (18)	0.0028 (15)
C10	0.027 (2)	0.032 (2)	0.040 (3)	0.0008 (17)	−0.003 (2)	−0.0026 (19)
C11	0.050 (3)	0.036 (3)	0.036 (3)	0.011 (2)	−0.002 (3)	−0.008 (2)
Cu2	0.0230 (4)	0.0224 (3)	0.0236 (4)	0.0011 (2)	0.0049 (3)	0.0016 (3)
Fe2	0.0186 (4)	0.0160 (3)	0.0179 (4)	−0.0002 (3)	0.0050 (3)	−0.0009 (3)
O2	0.016 (2)	0.035 (2)	0.042 (3)	−0.0022 (16)	0.0003 (19)	−0.0019 (19)
N11	0.034 (3)	0.026 (2)	0.023 (3)	0.006 (2)	0.006 (2)	0.0050 (19)
N12	0.021 (2)	0.0192 (18)	0.018 (2)	0.0023 (16)	0.0054 (19)	0.0003 (16)
N13	0.030 (3)	0.040 (3)	0.041 (3)	−0.006 (2)	0.004 (3)	−0.006 (2)
N14	0.031 (3)	0.026 (2)	0.044 (3)	0.0030 (19)	0.013 (2)	−0.003 (2)
N15	0.040 (3)	0.024 (2)	0.039 (3)	0.0061 (19)	0.015 (3)	0.005 (2)
N16	0.035 (2)	0.036 (2)	0.028 (2)	−0.0027 (17)	0.0111 (19)	−0.0032 (16)
N17	0.024 (2)	0.026 (2)	0.027 (2)	0.0036 (18)	0.008 (2)	0.0051 (18)
N18	0.025 (3)	0.032 (2)	0.032 (3)	−0.0036 (19)	−0.001 (2)	0.009 (2)
N19	0.042 (3)	0.026 (2)	0.039 (3)	0.0089 (18)	0.016 (2)	0.0073 (18)
N20	0.032 (3)	0.027 (2)	0.036 (3)	−0.0015 (17)	0.004 (2)	0.0051 (18)
C12	0.029 (3)	0.0151 (19)	0.022 (3)	0.0019 (19)	0.005 (2)	0.0004 (19)
C13	0.030 (3)	0.024 (2)	0.022 (3)	−0.006 (2)	0.008 (2)	−0.002 (2)
C14	0.029 (3)	0.022 (2)	0.022 (3)	−0.003 (2)	0.006 (2)	−0.0006 (19)
C15	0.021 (3)	0.023 (2)	0.019 (3)	0.0004 (18)	0.009 (2)	0.0044 (18)
C16	0.024 (2)	0.022 (2)	0.024 (3)	−0.0032 (16)	0.006 (2)	−0.0024 (17)
C17	0.043 (3)	0.0188 (19)	0.038 (3)	0.0012 (17)	0.010 (2)	0.0024 (17)
C18	0.042 (2)	0.031 (2)	0.032 (2)	−0.0077 (19)	−0.003 (2)	0.0015 (18)
C19	0.082 (6)	0.039 (3)	0.048 (4)	0.003 (3)	0.022 (4)	−0.016 (3)
C20	0.061 (3)	0.028 (2)	0.032 (2)	0.007 (2)	0.005 (2)	−0.0031 (19)
C21	0.058 (3)	0.022 (2)	0.043 (3)	−0.005 (2)	−0.008 (3)	0.0010 (19)
C22	0.066 (5)	0.044 (3)	0.050 (4)	0.013 (3)	0.009 (3)	−0.005 (3)
O1W	0.028 (2)	0.038 (2)	0.047 (3)	0.0050 (17)	0.005 (2)	0.0024 (19)
O2W	0.036 (2)	0.0306 (19)	0.042 (3)	0.0019 (16)	0.002 (2)	−0.0006 (17)

### (I) catena-Poly[[[bis[(R)-propane-1,2-diamine-κ2N,N']copper(II)]-µ-cyanido-κ2N:C-[tris(cyanido-κC)(nitroso-κN)iron(III)]-µ-cyanido-κ2C:N] monohydrate] Geometric parameters (Å, º)

**Table:**

Cu1—N10	1.998 (5)	Cu2—N20	2.013 (6)
Cu1—N8	2.001 (5)	Cu2—N18	2.017 (5)
Cu1—N7	2.003 (5)	Cu2—N11	2.290 (5)
Cu1—N9	2.026 (5)	Cu2—N3^ii^	3.112 (8)
Cu1—N1	2.333 (5)	Fe2—N12	1.646 (5)
Cu1—N13^i^	2.980 (9)	Fe2—C14	1.933 (5)
Fe1—N2	1.667 (5)	Fe2—C13	1.933 (6)
Fe1—C3	1.926 (5)	Fe2—C16	1.933 (6)
Fe1—C1	1.927 (6)	Fe2—C12	1.936 (6)
Fe1—C5	1.941 (6)	Fe2—C15	1.942 (5)
Fe1—C4	1.945 (5)	O2—N12	1.139 (6)
Fe1—C2	1.954 (6)	N11—C12	1.135 (8)
O1—N2	1.121 (6)	N13—C13	1.148 (8)
N1—C1	1.162 (8)	N14—C14	1.141 (8)
N3—C2	1.139 (8)	N15—C15	1.150 (7)
N4—C3	1.148 (7)	N16—C16	1.139 (8)
N5—C4	1.141 (7)	N17—C17	1.478 (7)
N6—C5	1.140 (8)	N17—H17X	0.8900
N7—C6	1.479 (7)	N17—H17Y	0.8900
N7—H7X	0.8900	N18—C18	1.457 (8)
N7—H7Y	0.8900	N18—H18X	0.8900
N8—C7	1.482 (6)	N18—H18Y	0.8900
N8—H8X	0.8900	N19—C20	1.498 (8)
N8—H8Y	0.8900	N19—H19X	0.8900
N9—C9	1.484 (6)	N19—H19Y	0.8900
N9—H9X	0.8900	N20—C21	1.475 (8)
N9—H9Y	0.8900	N20—H20X	0.8900
N10—C10	1.454 (8)	N20—H20Y	0.8900
N10—H10X	0.8900	C17—C18	1.497 (7)
N10—H10Y	0.8900	C17—C19	1.534 (9)
C6—C7	1.506 (7)	C17—H17	1.0000
C6—C8	1.519 (9)	C18—H18A	0.9900
C6—H6	1.0000	C18—H18B	0.9900
C7—H7A	0.9900	C19—H19A	0.9800
C7—H7B	0.9900	C19—H19B	0.9800
C8—H8A	0.9800	C19—H19C	0.9800
C8—H8B	0.9800	C20—C21	1.465 (8)
C8—H8C	0.9800	C20—C22	1.513 (9)
C9—C10	1.511 (6)	C20—H20	1.0000
C9—C11	1.512 (8)	C21—H21A	0.9900
C9—H9	1.0000	C21—H21B	0.9900
C10—H10A	0.9900	C22—H22A	0.9800
C10—H10B	0.9900	C22—H22B	0.9800
C11—H11A	0.9800	C22—H22C	0.9800
C11—H11B	0.9800	O1W—H1WA	0.82 (3)
C11—H11C	0.9800	O1W—H1WB	0.84 (3)
Cu2—N19	2.006 (6)	O2W—H2WA	0.83 (3)
Cu2—N17	2.009 (5)	O2W—H2WB	0.80 (3)
			
N10—Cu1—N8	175.5 (2)	N19—Cu2—N20	84.8 (2)
N10—Cu1—N7	95.2 (2)	N17—Cu2—N20	92.6 (2)
N8—Cu1—N7	85.0 (2)	N19—Cu2—N18	97.6 (3)
N10—Cu1—N9	84.3 (2)	N17—Cu2—N18	84.9 (2)
N8—Cu1—N9	95.2 (2)	N20—Cu2—N18	163.6 (2)
N7—Cu1—N9	175.3 (2)	N19—Cu2—N11	89.6 (2)
N10—Cu1—N1	94.7 (2)	N17—Cu2—N11	90.9 (2)
N8—Cu1—N1	89.8 (2)	N20—Cu2—N11	101.0 (2)
N7—Cu1—N1	91.7 (2)	N18—Cu2—N11	95.2 (2)
N9—Cu1—N1	93.0 (2)	N12—Fe2—C14	95.8 (2)
N2—Fe1—C3	94.0 (2)	N12—Fe2—C13	178.5 (2)
N2—Fe1—C1	94.7 (3)	C14—Fe2—C13	82.7 (3)
C3—Fe1—C1	88.7 (3)	N12—Fe2—C16	94.3 (2)
N2—Fe1—C5	95.8 (3)	C14—Fe2—C16	87.5 (2)
C3—Fe1—C5	88.8 (3)	C13—Fe2—C16	85.6 (3)
C1—Fe1—C5	169.4 (2)	N12—Fe2—C12	94.3 (2)
N2—Fe1—C4	93.7 (2)	C14—Fe2—C12	88.7 (2)
C3—Fe1—C4	172.2 (3)	C13—Fe2—C12	85.7 (3)
C1—Fe1—C4	91.5 (2)	C16—Fe2—C12	170.9 (2)
C5—Fe1—C4	89.6 (3)	N12—Fe2—C15	95.0 (2)
N2—Fe1—C2	177.8 (2)	C14—Fe2—C15	169.2 (3)
C3—Fe1—C2	84.1 (2)	C13—Fe2—C15	86.5 (2)
C1—Fe1—C2	84.3 (3)	C16—Fe2—C15	90.6 (2)
C5—Fe1—C2	85.2 (3)	C12—Fe2—C15	91.6 (2)
C4—Fe1—C2	88.2 (2)	C12—N11—Cu2	150.5 (5)
C1—N1—Cu1	152.2 (5)	O2—N12—Fe2	179.6 (5)
O1—N2—Fe1	178.7 (5)	C17—N17—Cu2	109.0 (3)
C6—N7—Cu1	109.8 (4)	C17—N17—H17X	109.9
C6—N7—H7X	110.1	Cu2—N17—H17X	109.9
Cu1—N7—H7X	109.6	C17—N17—H17Y	110.0
C6—N7—H7Y	109.6	Cu2—N17—H17Y	109.8
Cu1—N7—H7Y	109.6	H17X—N17—H17Y	108.2
H7X—N7—H7Y	108.2	C18—N18—Cu2	107.7 (4)
C7—N8—Cu1	108.0 (3)	C18—N18—H18X	110.0
C7—N8—H8X	109.6	Cu2—N18—H18X	109.8
Cu1—N8—H8X	109.9	C18—N18—H18Y	110.5
C7—N8—H8Y	110.4	Cu2—N18—H18Y	110.4
Cu1—N8—H8Y	110.4	H18X—N18—H18Y	108.4
H8X—N8—H8Y	108.5	C20—N19—Cu2	106.7 (4)
C9—N9—Cu1	109.2 (4)	C20—N19—H19X	110.6
C9—N9—H9X	110.1	Cu2—N19—H19X	110.4
Cu1—N9—H9X	109.8	C20—N19—H19Y	110.3
C9—N9—H9Y	109.7	Cu2—N19—H19Y	110.2
Cu1—N9—H9Y	109.7	H19X—N19—H19Y	108.5
H9X—N9—H9Y	108.3	C21—N20—Cu2	108.4 (4)
C10—N10—Cu1	109.1 (4)	C21—N20—H20X	110.1
C10—N10—H10X	109.5	Cu2—N20—H20X	109.9
Cu1—N10—H10X	109.7	C21—N20—H20Y	110.0
C10—N10—H10Y	110.1	Cu2—N20—H20Y	110.1
Cu1—N10—H10Y	110.0	H20X—N20—H20Y	108.4
H10X—N10—H10Y	108.3	N11—C12—Fe2	176.0 (5)
N1—C1—Fe1	176.2 (5)	N13—C13—Fe2	177.3 (5)
N3—C2—Fe1	176.4 (6)	N14—C14—Fe2	178.3 (6)
N4—C3—Fe1	178.1 (6)	N15—C15—Fe2	176.9 (5)
N5—C4—Fe1	175.3 (6)	N16—C16—Fe2	175.4 (5)
N6—C5—Fe1	178.6 (6)	N17—C17—C18	107.4 (4)
N7—C6—C7	106.9 (4)	N17—C17—C19	112.0 (5)
N7—C6—C8	112.3 (6)	C18—C17—C19	111.6 (5)
C7—C6—C8	110.5 (5)	N17—C17—H17	108.6
N7—C6—H6	109.0	C18—C17—H17	108.6
C7—C6—H6	109.0	C19—C17—H17	108.6
C8—C6—H6	109.0	N18—C18—C17	110.3 (4)
N8—C7—C6	109.0 (4)	N18—C18—H18A	109.6
N8—C7—H7A	109.9	C17—C18—H18A	109.6
C6—C7—H7A	109.9	N18—C18—H18B	109.6
N8—C7—H7B	109.9	C17—C18—H18B	109.6
C6—C7—H7B	109.9	H18A—C18—H18B	108.1
H7A—C7—H7B	108.3	C17—C19—H19A	109.5
C6—C8—H8A	109.5	C17—C19—H19B	109.5
C6—C8—H8B	109.5	H19A—C19—H19B	109.5
H8A—C8—H8B	109.5	C17—C19—H19C	109.5
C6—C8—H8C	109.5	H19A—C19—H19C	109.5
H8A—C8—H8C	109.5	H19B—C19—H19C	109.5
H8B—C8—H8C	109.5	C21—C20—N19	107.5 (4)
N9—C9—C10	106.6 (4)	C21—C20—C22	110.8 (5)
N9—C9—C11	112.1 (5)	N19—C20—C22	113.9 (5)
C10—C9—C11	113.8 (4)	C21—C20—H20	108.2
N9—C9—H9	108.1	N19—C20—H20	108.2
C10—C9—H9	108.1	C22—C20—H20	108.2
C11—C9—H9	108.1	C20—C21—N20	108.6 (4)
N10—C10—C9	109.1 (4)	C20—C21—H21A	110.0
N10—C10—H10A	109.9	N20—C21—H21A	110.0
C9—C10—H10A	109.9	C20—C21—H21B	110.0
N10—C10—H10B	109.9	N20—C21—H21B	110.0
C9—C10—H10B	109.9	H21A—C21—H21B	108.4
H10A—C10—H10B	108.3	C20—C22—H22A	109.5
C9—C11—H11A	109.5	C20—C22—H22B	109.5
C9—C11—H11B	109.5	H22A—C22—H22B	109.5
H11A—C11—H11B	109.5	C20—C22—H22C	109.5
C9—C11—H11C	109.5	H22A—C22—H22C	109.5
H11A—C11—H11C	109.5	H22B—C22—H22C	109.5
H11B—C11—H11C	109.5	H1WA—O1W—H1WB	105 (4)
N19—Cu2—N17	177.4 (2)	H2WA—O2W—H2WB	114 (4)
			
Cu1—N7—C6—C7	37.2 (5)	Cu2—N17—C17—C18	37.3 (5)
Cu1—N7—C6—C8	158.6 (5)	Cu2—N17—C17—C19	160.2 (5)
Cu1—N8—C7—C6	39.8 (5)	Cu2—N18—C18—C17	38.5 (5)
N7—C6—C7—N8	−50.9 (6)	N17—C17—C18—N18	−50.7 (5)
C8—C6—C7—N8	−173.3 (6)	C19—C17—C18—N18	−173.9 (5)
Cu1—N9—C9—C10	37.3 (5)	Cu2—N19—C20—C21	44.0 (5)
Cu1—N9—C9—C11	162.4 (4)	Cu2—N19—C20—C22	167.2 (5)
Cu1—N10—C10—C9	40.4 (5)	N19—C20—C21—N20	−54.1 (6)
N9—C9—C10—N10	−51.3 (5)	C22—C20—C21—N20	−179.1 (5)
C11—C9—C10—N10	−175.4 (5)	Cu2—N20—C21—C20	37.2 (5)

Symmetry codes: (i) −*x*+1, *y*−1/2, −*z*+1; (ii) −*x*, *y*+1/2, −*z*.

### (I) catena-Poly[[[bis[(R)-propane-1,2-diamine-κ2N,N']copper(II)]-µ-cyanido-κ2N:C-[tris(cyanido-κC)(nitroso-κN)iron(III)]-µ-cyanido-κ2C:N] monohydrate] Hydrogen-bond geometry (Å, º)

**Table:**

*D*—H···*A*	*D*—H	H···*A*	*D*···*A*	*D*—H···*A*
N7—H7*X*···N15^iii^	0.89	2.39	3.257 (8)	166
N7—H7*Y*···N14^iv^	0.89	2.12	3.008 (8)	173
N8—H8*X*···N14^i^	0.89	2.27	3.120 (8)	160
N8—H8*Y*···N15	0.89	2.45	3.209 (7)	144
N9—H9*X*···O1*W*	0.89	2.52	3.207 (7)	135
N9—H9*Y*···N16^i^	0.89	2.39	3.157 (7)	144
N10—H10*X*···N16^iv^	0.89	2.52	3.189 (7)	132
N10—H10*Y*···O1*W*^iii^	0.89	2.11	2.962 (7)	159
C11—H11*A*···N16^i^	0.98	2.68	3.427 (9)	134
N17—H17*X*···N4^v^	0.89	2.22	3.051 (8)	155
N17—H17*Y*···N5^vi^	0.89	2.32	3.197 (7)	169
N18—H18*X*···N5	0.89	2.37	3.224 (8)	161
N18—H18*Y*···N4^ii^	0.89	2.27	3.080 (8)	151
N19—H19*X*···N6^ii^	0.89	2.44	3.295 (8)	160
N19—H19*Y*···O2*W*^iii^	0.89	2.30	3.142 (8)	159
N20—H20*X*···O2*W*	0.89	2.11	2.990 (8)	172
O1*W*—H1*WB*···N15	0.84 (3)	2.11 (3)	2.928 (7)	163 (6)
O2*W*—H2*WA*···N13	0.83 (3)	2.65 (4)	3.419 (9)	155 (6)
O2*W*—H2*WB*···N20	0.80 (3)	2.37 (5)	2.990 (8)	135 (6)

Symmetry codes: (i) −*x*+1, *y*−1/2, −*z*+1; (ii) −*x*, *y*+1/2, −*z*; (iii) *x*−1, *y*, *z*; (iv) −*x*, *y*−1/2, −*z*+1; (v) −*x*+1, *y*+1/2, −*z*; (vi) *x*+1, *y*, *z*.

### (II) Poly[[hexa-µ-cyanido-κ12C:N-hexacyanido-κ6C-hexakis[(R)-propane-1,2-diamine-κ2N,N']dichromiun(III)tricopper(II)] pentahydrate] Crystal data

**Table:**

[Cr_2_Cu_3_(CN)_12_(C_3_H_10_N_2_)_6_]·5H_2_O	*F*(000) = 1186
*M**_r_* = 1141.72	*D*_x_ = 1.407 Mg m^−^^3^
Monoclinic, *P*2_1_	Mo *K*α radiation, λ = 0.71073 Å
*a* = 10.1474 (10) Å	Cell parameters from 21484 reflections
*b* = 17.6136 (10) Å	θ = 0.1–24.9°
*c* = 15.5376 (14) Å	µ = 1.61 mm^−^^1^
β = 103.973 (11)°	*T* = 173 K
*V* = 2694.9 (4) Å^3^	Block, blue
*Z* = 2	0.40 × 0.30 × 0.30 mm

### (II) Poly[[hexa-µ-cyanido-κ12C:N-hexacyanido-κ6C-hexakis[(R)-propane-1,2-diamine-κ2N,N']dichromiun(III)tricopper(II)] pentahydrate] Data collection

**Table:**

Stoe IPDS 2 diffractometer	10308 independent reflections
Radiation source: fine-focus sealed tube	4948 reflections with *I* > 2σ(*I*)
Plane graphite monochromator	*R*_int_ = 0.085
φ + ω scans	θ_max_ = 26.1°, θ_min_ = 2.1°
Absorption correction: multi-scan (MULABS in PLATON; Spek, 2009)	*h* = −12→12
*T*_min_ = 0.583, *T*_max_ = 0.678	*k* = −21→21
21461 measured reflections	*l* = −18→19

### (II) Poly[[hexa-µ-cyanido-κ12C:N-hexacyanido-κ6C-hexakis[(R)-propane-1,2-diamine-κ2N,N']dichromiun(III)tricopper(II)] pentahydrate] Refinement

**Table:**

Refinement on *F*^2^	Hydrogen site location: mixed
Least-squares matrix: full	H atoms treated by a mixture of independent and constrained refinement
*R*[*F*^2^ > 2σ(*F*^2^)] = 0.052	*w* = 1/[σ^2^(*F*_o_^2^) + (0.0562*P*)^2^] where *P* = (*F*_o_^2^ + 2*F*_c_^2^)/3
*wR*(*F*^2^) = 0.131	(Δ/σ)_max_ < 0.001
*S* = 0.79	Δρ_max_ = 0.61 e Å^−^^3^
10308 reflections	Δρ_min_ = −1.05 e Å^−^^3^
594 parameters	Extinction correction: SHELXL2014 (Sheldrick, 2015), Fc^*^=kFc[1+0.001xFc^2^λ^3^/sin(2θ)]^-1/4^
14 restraints	Extinction coefficient: 0.0020 (3)
Primary atom site location: structure-invariant direct methods	Absolute structure: Flack *x* determined using 1754 quotients [(*I*^+^)-(*I*^-^)]/[(*I*^+^)+(*I*^-^)] (Parsons *et al.*, 2013)
Secondary atom site location: difference Fourier map	Absolute structure parameter: 0.00 (3)

### (II) Poly[[hexa-µ-cyanido-κ12C:N-hexacyanido-κ6C-hexakis[(R)-propane-1,2-diamine-κ2N,N']dichromiun(III)tricopper(II)] pentahydrate] Special details

**Table:**

Geometry. All e.s.d.'s (except the e.s.d. in the dihedral angle between two l.s. planes) are estimated using the full covariance matrix. The cell e.s.d.'s are taken into account individually in the estimation of e.s.d.'s in distances, angles and torsion angles; correlations between e.s.d.'s in cell parameters are only used when they are defined by crystal symmetry. An approximate (isotropic) treatment of cell e.s.d.'s is used for estimating e.s.d.'s involving l.s. planes.

### (II) Poly[[hexa-µ-cyanido-κ12C:N-hexacyanido-κ6C-hexakis[(R)-propane-1,2-diamine-κ2N,N']dichromiun(III)tricopper(II)] pentahydrate] Fractional atomic coordinates and isotropic or equivalent isotropic displacement parameters (Å^2^)

**Table:**

	*x*	*y*	*z*	*U*_iso_*/*U*_eq_	Occ. (<1)
Cu1	0.51532 (17)	−0.16435 (9)	0.17076 (10)	0.0541 (5)	
Cu2	1.02528 (15)	0.55090 (9)	0.33881 (9)	0.0478 (5)	
Cu3	0.73882 (16)	0.20223 (11)	0.24976 (12)	0.0445 (4)	
Cr1	0.48267 (17)	0.07601 (10)	−0.02939 (11)	0.0342 (5)	
Cr2	0.99071 (17)	0.31948 (10)	0.52845 (11)	0.0338 (5)	
N1	0.5975 (11)	0.2178 (7)	0.0977 (7)	0.051 (3)	
N2	0.5099 (12)	−0.0213 (7)	0.1496 (8)	0.058 (3)	
N3	0.4666 (13)	0.1845 (8)	−0.1992 (8)	0.065 (4)	
N4	0.4687 (12)	−0.1554 (8)	0.2882 (7)	0.068 (4)	
H4X	0.5307	−0.1793	0.3296	0.081*	
H4Y	0.4676	−0.1068	0.3035	0.081*	
N5	0.3166 (13)	−0.1839 (9)	0.1257 (8)	0.085 (4)	
H5X	0.2834	−0.1534	0.0799	0.102*	
H5Y	0.3041	−0.2317	0.1067	0.102*	
N6	0.7170 (11)	−0.1651 (7)	0.2205 (7)	0.060 (3)	
H6X	0.7447	−0.1195	0.2423	0.071*	
H6Y	0.7387	−0.1988	0.2643	0.071*	
N7	0.5640 (11)	−0.1689 (7)	0.0541 (7)	0.057 (3)	
H7X	0.5495	−0.2157	0.0321	0.068*	
H7Y	0.5112	−0.1372	0.0163	0.068*	
N8	0.1712 (12)	0.1231 (8)	−0.0411 (10)	0.065 (4)	
N9	0.3824 (11)	−0.0737 (7)	−0.1487 (8)	0.057 (3)	
N10	0.7910 (13)	0.0303 (7)	−0.0181 (9)	0.063 (4)	
N11	0.8801 (12)	0.1718 (7)	0.4122 (8)	0.059 (4)	
N12	1.0084 (10)	0.4105 (7)	0.3515 (8)	0.047 (3)	
N13	0.9876 (12)	0.2109 (8)	0.6935 (8)	0.059 (3)	
N14	0.8281 (10)	0.5536 (7)	0.2720 (7)	0.052 (3)	
H14X	0.8091	0.5980	0.2443	0.063*	
H14Y	0.8114	0.5169	0.2315	0.063*	
N15	0.9496 (11)	0.5592 (7)	0.4455 (7)	0.057 (3)	
H15X	0.9528	0.5141	0.4717	0.068*	
H15Y	0.9992	0.5916	0.4840	0.068*	
N16	1.2223 (12)	0.5567 (8)	0.4047 (9)	0.081 (4)	
H16X	1.2309	0.5868	0.4518	0.098*	
H16Y	1.2520	0.5107	0.4237	0.098*	
N17	1.1002 (11)	0.5557 (7)	0.2339 (8)	0.067 (3)	
H17X	1.0659	0.5180	0.1971	0.081*	
H17Y	1.0762	0.5994	0.2057	0.081*	
N18	1.0917 (12)	0.4670 (7)	0.6493 (8)	0.056 (3)	
N19	1.3045 (12)	0.2783 (7)	0.5541 (8)	0.056 (3)	
N20	0.6777 (12)	0.3732 (7)	0.4901 (8)	0.060 (3)	
N21	0.8943 (12)	0.2578 (8)	0.2240 (8)	0.066 (4)	
H21X	0.8677	0.2831	0.1731	0.079*	
H21Y	0.9267	0.2910	0.2670	0.079*	
N22	0.8271 (12)	0.1100 (8)	0.2148 (8)	0.063 (4)	
H22X	0.8629	0.0825	0.2629	0.075*	
H22Y	0.7650	0.0817	0.1785	0.075*	
N23	0.6505 (12)	0.2948 (7)	0.2834 (7)	0.059 (3)	
H23X	0.6979	0.3120	0.3356	0.071*	
H23Y	0.6481	0.3310	0.2431	0.071*	
N24	0.5846 (11)	0.1492 (8)	0.2809 (8)	0.059 (3)	
H24X	0.5273	0.1325	0.2318	0.071*	
H24Y	0.6150	0.1093	0.3151	0.071*	
C1	0.5538 (12)	0.1684 (8)	0.0494 (9)	0.042 (3)	
C2	0.4959 (12)	0.0149 (8)	0.0868 (9)	0.042 (3)	
C3	0.4714 (13)	0.1428 (8)	−0.1401 (10)	0.046 (3)	
C4	0.336 (2)	−0.1892 (14)	0.2814 (12)	0.111 (4)	
H4A	0.2984	−0.1705	0.3307	0.133*	
H4B	0.3454	−0.2450	0.2871	0.133*	
C5	0.241 (2)	−0.1708 (17)	0.1968 (12)	0.111 (4)	
H5	0.1592	−0.2044	0.1870	0.133*	
C6	0.199 (2)	−0.0890 (13)	0.1930 (13)	0.111 (4)	
H6A	0.1438	−0.0772	0.1336	0.166*	
H6B	0.2802	−0.0567	0.2061	0.166*	
H6C	0.1459	−0.0795	0.2368	0.166*	
C7	0.7840 (14)	−0.1851 (9)	0.1491 (9)	0.064 (4)	
H7A	0.7844	−0.2409	0.1417	0.076*	
H7B	0.8793	−0.1672	0.1647	0.076*	
C8	0.7079 (15)	−0.1482 (9)	0.0637 (9)	0.064 (4)	
H8	0.7168	−0.0919	0.0706	0.077*	
C9	0.7626 (17)	−0.1715 (12)	−0.0140 (12)	0.083 (5)	
H9A	0.7148	−0.1437	−0.0669	0.124*	
H9B	0.7489	−0.2262	−0.0242	0.124*	
H9C	0.8599	−0.1600	−0.0014	0.124*	
C10	0.2848 (15)	0.1048 (8)	−0.0364 (10)	0.048 (4)	
C11	0.4177 (13)	−0.0199 (9)	−0.1051 (9)	0.045 (3)	
C12	0.6798 (14)	0.0468 (9)	−0.0239 (8)	0.045 (3)	
C13	0.9223 (11)	0.2246 (8)	0.4512 (7)	0.038 (3)	
C14	1.0025 (12)	0.3780 (8)	0.4143 (10)	0.040 (3)	
C15	0.9862 (12)	0.2523 (8)	0.6373 (9)	0.044 (3)	
C16	0.7459 (13)	0.5435 (9)	0.3339 (9)	0.058 (4)	
H16A	0.7401	0.4888	0.3472	0.070*	
H16B	0.6528	0.5622	0.3077	0.070*	
C17	0.8058 (14)	0.5863 (9)	0.4186 (9)	0.059 (4)	
H17	0.8077	0.6413	0.4032	0.071*	
C18	0.7343 (17)	0.5794 (11)	0.4909 (11)	0.080 (5)	
H18A	0.7916	0.6002	0.5459	0.120*	
H18B	0.7153	0.5258	0.4997	0.120*	
H18C	0.6487	0.6077	0.4750	0.120*	
C19	1.3002 (18)	0.5853 (13)	0.3487 (14)	0.108 (7)	
H19A	1.3973	0.5731	0.3732	0.130*	
H19B	1.2905	0.6411	0.3440	0.130*	
C20	1.2512 (16)	0.5496 (11)	0.2590 (14)	0.089 (6)	
H20	1.2880	0.5796	0.2155	0.107*	
C21	1.2885 (19)	0.4656 (13)	0.2508 (12)	0.112 (7)	
H21C	1.3869	0.4591	0.2722	0.169*	
H21B	1.2603	0.4499	0.1886	0.169*	
H21A	1.2421	0.4342	0.2865	0.169*	
C22	1.0531 (12)	0.4149 (8)	0.6067 (8)	0.041 (3)	
C23	1.1914 (14)	0.2903 (7)	0.5443 (8)	0.041 (3)	
C24	0.7905 (14)	0.3520 (8)	0.5069 (8)	0.044 (3)	
C25	1.0019 (17)	0.2019 (12)	0.2175 (12)	0.087 (3)	
H25A	1.0575	0.1892	0.2773	0.104*	
H25B	1.0623	0.2234	0.1823	0.104*	
C26	0.9301 (17)	0.1304 (12)	0.1719 (12)	0.087 (3)	
H26	0.8884	0.1416	0.1080	0.104*	
C27	1.0398 (15)	0.0766 (11)	0.1785 (11)	0.087 (3)	
H27A	1.0745	0.0616	0.2406	0.130*	
H27B	1.1130	0.1002	0.1566	0.130*	
H27C	1.0063	0.0317	0.1427	0.130*	
C28	0.5164 (19)	0.2764 (10)	0.2887 (11)	0.084 (6)	
H28A	0.4552	0.2768	0.2285	0.101*	
H28B	0.4836	0.3149	0.3250	0.101*	
C29	0.5137 (17)	0.2017 (11)	0.3283 (11)	0.078 (5)	
H29	0.5714	0.2055	0.3902	0.094*	
C30	0.3779 (18)	0.1714 (12)	0.3362 (13)	0.111 (7)	
H30A	0.3882	0.1185	0.3564	0.166*	
H30B	0.3130	0.1739	0.2782	0.166*	
H30C	0.3444	0.2020	0.3791	0.166*	
O1W	0.4077 (9)	0.4169 (5)	0.4832 (7)	0.065 (3)	
H1WA	0.387 (13)	0.379 (6)	0.511 (10)	0.098*	
H1WB	0.485 (8)	0.407 (8)	0.475 (10)	0.098*	
O2W	0.9673 (10)	0.4758 (7)	0.0537 (9)	0.087 (3)	
H2WA	1.028 (14)	0.477 (10)	0.025 (11)	0.130*	
H2WB	0.931 (17)	0.432 (5)	0.046 (13)	0.130*	
O3W	0.6417 (16)	0.0077 (9)	0.3872 (9)	0.116 (5)	
H3WA	0.726 (5)	−0.001 (15)	0.404 (14)	0.174*	
H3WB	0.606 (18)	−0.012 (15)	0.426 (12)	0.174*	
O4W	0.1006 (11)	−0.3169 (7)	0.0730 (9)	0.095 (4)	
H4WA	0.114 (15)	−0.338 (12)	0.027 (8)	0.143*	
H4WB	0.016 (4)	−0.314 (12)	0.066 (11)	0.143*	
O5WA	0.815 (4)	0.362 (3)	0.064 (3)	0.056 (14)	0.43 (17)
O5WB	0.857 (12)	0.327 (7)	0.052 (4)	0.13 (4)	0.57 (17)

### (II) Poly[[hexa-µ-cyanido-κ12C:N-hexacyanido-κ6C-hexakis[(R)-propane-1,2-diamine-κ2N,N']dichromiun(III)tricopper(II)] pentahydrate] Atomic displacement parameters (Å^2^)

**Table:**

	*U*^11^	*U*^22^	*U*^33^	*U*^12^	*U*^13^	*U*^23^
Cu1	0.0756 (11)	0.0442 (11)	0.0307 (8)	−0.0028 (8)	−0.0101 (7)	−0.0017 (7)
Cu2	0.0593 (10)	0.0442 (10)	0.0313 (8)	−0.0020 (8)	−0.0058 (7)	−0.0040 (7)
Cu3	0.0575 (9)	0.0389 (8)	0.0291 (6)	−0.0067 (7)	−0.0051 (6)	0.0014 (6)
Cr1	0.0428 (10)	0.0268 (12)	0.0283 (10)	−0.0036 (8)	−0.0008 (8)	0.0002 (9)
Cr2	0.0452 (10)	0.0256 (12)	0.0250 (10)	−0.0010 (8)	−0.0026 (8)	0.0009 (8)
N1	0.068 (7)	0.043 (8)	0.024 (5)	0.001 (6)	−0.023 (5)	0.002 (5)
N2	0.102 (10)	0.032 (7)	0.037 (7)	0.000 (6)	0.012 (6)	0.007 (6)
N3	0.106 (10)	0.040 (8)	0.045 (7)	0.012 (7)	0.008 (7)	0.012 (6)
N4	0.080 (8)	0.064 (9)	0.047 (7)	0.013 (7)	−0.008 (6)	−0.002 (6)
N5	0.097 (9)	0.085 (11)	0.060 (8)	−0.029 (9)	−0.007 (7)	−0.007 (8)
N6	0.085 (8)	0.038 (7)	0.040 (6)	−0.003 (6)	−0.016 (6)	0.005 (6)
N7	0.078 (8)	0.040 (7)	0.040 (6)	−0.005 (7)	−0.010 (5)	0.004 (6)
N8	0.049 (7)	0.052 (8)	0.088 (10)	−0.002 (6)	0.007 (6)	0.004 (7)
N9	0.064 (7)	0.043 (8)	0.049 (7)	−0.003 (6)	−0.015 (6)	−0.010 (6)
N10	0.064 (8)	0.042 (8)	0.084 (10)	−0.008 (6)	0.018 (7)	−0.002 (7)
N11	0.086 (8)	0.028 (7)	0.045 (7)	−0.001 (6)	−0.023 (6)	−0.012 (6)
N12	0.061 (7)	0.041 (8)	0.036 (7)	−0.003 (5)	0.008 (5)	0.001 (6)
N13	0.089 (8)	0.050 (8)	0.041 (7)	0.005 (7)	0.020 (6)	0.020 (7)
N14	0.070 (7)	0.038 (7)	0.041 (6)	−0.005 (6)	−0.003 (5)	−0.001 (6)
N15	0.079 (7)	0.029 (7)	0.046 (7)	−0.001 (6)	−0.016 (5)	−0.008 (6)
N16	0.067 (8)	0.062 (9)	0.099 (11)	−0.003 (7)	−0.010 (7)	0.010 (8)
N17	0.081 (8)	0.053 (8)	0.064 (8)	0.009 (7)	0.007 (6)	0.012 (6)
N18	0.077 (8)	0.026 (7)	0.054 (7)	−0.006 (6)	−0.007 (6)	−0.010 (6)
N19	0.058 (8)	0.050 (8)	0.056 (8)	−0.003 (6)	0.009 (6)	−0.001 (6)
N20	0.056 (7)	0.060 (8)	0.061 (8)	0.011 (6)	0.006 (6)	0.013 (6)
N21	0.097 (9)	0.055 (8)	0.032 (7)	−0.029 (7)	−0.008 (6)	0.000 (6)
N22	0.069 (8)	0.063 (9)	0.039 (7)	−0.005 (6)	−0.018 (6)	0.005 (6)
N23	0.078 (9)	0.061 (9)	0.031 (6)	0.003 (6)	0.000 (6)	0.005 (6)
N24	0.079 (8)	0.060 (9)	0.032 (6)	−0.005 (6)	−0.001 (6)	0.001 (6)
C1	0.047 (7)	0.036 (8)	0.035 (7)	0.005 (6)	−0.008 (6)	0.018 (7)
C2	0.045 (7)	0.041 (9)	0.037 (8)	−0.006 (6)	0.005 (6)	−0.006 (7)
C3	0.054 (8)	0.028 (8)	0.049 (9)	−0.003 (6)	−0.001 (6)	−0.011 (7)
C4	0.123 (9)	0.132 (11)	0.079 (7)	0.000 (9)	0.028 (6)	−0.008 (9)
C5	0.123 (9)	0.132 (11)	0.079 (7)	0.000 (9)	0.028 (6)	−0.008 (9)
C6	0.123 (9)	0.132 (11)	0.079 (7)	0.000 (9)	0.028 (6)	−0.008 (9)
C7	0.072 (9)	0.055 (10)	0.054 (9)	0.005 (8)	−0.004 (7)	0.009 (8)
C8	0.093 (11)	0.049 (10)	0.048 (8)	−0.009 (8)	0.014 (7)	0.007 (7)
C9	0.112 (12)	0.058 (11)	0.086 (12)	0.002 (10)	0.039 (10)	0.012 (10)
C10	0.068 (9)	0.027 (8)	0.046 (9)	−0.009 (7)	0.004 (7)	0.001 (6)
C11	0.051 (8)	0.037 (9)	0.042 (8)	0.011 (6)	0.002 (6)	0.015 (7)
C12	0.047 (8)	0.046 (9)	0.038 (7)	−0.016 (7)	0.005 (6)	−0.004 (7)
C13	0.048 (7)	0.037 (9)	0.020 (6)	0.013 (6)	−0.011 (5)	0.009 (6)
C14	0.045 (7)	0.023 (8)	0.046 (9)	0.009 (5)	0.000 (6)	−0.005 (7)
C15	0.055 (8)	0.040 (9)	0.036 (8)	−0.005 (6)	0.007 (6)	−0.015 (7)
C16	0.045 (8)	0.053 (10)	0.069 (10)	0.002 (7)	−0.003 (7)	0.009 (8)
C17	0.080 (10)	0.042 (9)	0.061 (9)	0.008 (7)	0.030 (8)	0.002 (7)
C18	0.108 (13)	0.061 (12)	0.081 (12)	0.011 (10)	0.045 (10)	−0.002 (10)
C19	0.080 (13)	0.104 (16)	0.121 (17)	−0.026 (11)	−0.015 (12)	0.039 (14)
C20	0.061 (10)	0.077 (13)	0.144 (18)	0.011 (10)	0.050 (11)	0.026 (13)
C21	0.105 (14)	0.15 (2)	0.084 (13)	0.036 (14)	0.020 (10)	0.028 (13)
C22	0.055 (8)	0.033 (8)	0.030 (7)	0.010 (6)	0.000 (6)	0.013 (6)
C23	0.053 (8)	0.033 (8)	0.028 (7)	−0.010 (6)	−0.007 (6)	0.008 (6)
C24	0.068 (9)	0.031 (8)	0.032 (7)	0.000 (7)	0.007 (6)	0.001 (6)
C25	0.077 (7)	0.105 (9)	0.074 (7)	0.009 (6)	0.012 (5)	0.006 (6)
C26	0.077 (7)	0.105 (9)	0.074 (7)	0.009 (6)	0.012 (5)	0.006 (6)
C27	0.077 (7)	0.105 (9)	0.074 (7)	0.009 (6)	0.012 (5)	0.006 (6)
C28	0.108 (15)	0.086 (14)	0.059 (10)	0.040 (11)	0.023 (9)	−0.010 (9)
C29	0.087 (12)	0.087 (14)	0.065 (10)	0.012 (11)	0.028 (9)	0.012 (10)
C30	0.104 (14)	0.135 (18)	0.115 (16)	−0.022 (13)	0.066 (12)	−0.006 (13)
O1W	0.053 (6)	0.052 (6)	0.092 (8)	0.005 (5)	0.018 (5)	0.015 (5)
O2W	0.047 (7)	0.091 (9)	0.123 (10)	0.004 (6)	0.020 (6)	0.018 (8)
O3W	0.155 (13)	0.102 (11)	0.098 (10)	0.047 (10)	0.046 (9)	0.047 (8)
O4W	0.090 (8)	0.076 (9)	0.131 (11)	−0.023 (7)	0.050 (7)	−0.041 (7)
O5WA	0.06 (2)	0.04 (2)	0.057 (17)	0.009 (18)	−0.007 (11)	−0.006 (16)
O5WB	0.17 (5)	0.11 (6)	0.09 (2)	−0.10 (5)	−0.04 (3)	0.06 (3)

### (II) Poly[[hexa-µ-cyanido-κ12C:N-hexacyanido-κ6C-hexakis[(R)-propane-1,2-diamine-κ2N,N']dichromiun(III)tricopper(II)] pentahydrate] Geometric parameters (Å, º)

**Table:**

Cu1—N7	1.992 (12)	N21—H21Y	0.8900
Cu1—N5	1.998 (12)	N22—C26	1.42 (2)
Cu1—N4	1.999 (12)	N22—H22X	0.8900
Cu1—N6	2.006 (10)	N22—H22Y	0.8900
Cu1—N2	2.540 (12)	N23—C28	1.42 (2)
Cu1—N3^i^	2.698 (14)	N23—H23X	0.8900
Cu2—N17	1.960 (12)	N23—H23Y	0.8900
Cu2—N15	1.993 (12)	N24—C29	1.47 (2)
Cu2—N16	2.017 (11)	N24—H24X	0.8900
Cu2—N14	2.020 (10)	N24—H24Y	0.8900
Cu2—N12	2.490 (12)	C4—C5	1.47 (2)
Cu2—N13^ii^	2.860 (14)	C4—H4A	0.9900
Cu3—N21	1.978 (12)	C4—H4B	0.9900
Cu3—N24	1.981 (12)	C5—C6	1.50 (3)
Cu3—N23	1.990 (13)	C5—H5	1.0000
Cu3—N22	1.993 (14)	C6—H6A	0.9800
Cu3—N1	2.465 (9)	C6—H6B	0.9800
Cu3—N11	2.639 (12)	C6—H6C	0.9800
Cr1—C12	2.047 (15)	C7—C8	1.512 (18)
Cr1—C10	2.049 (16)	C7—H7A	0.9900
Cr1—C1	2.060 (14)	C7—H7B	0.9900
Cr1—C3	2.065 (16)	C8—C9	1.50 (2)
Cr1—C11	2.073 (16)	C8—H8	1.0000
Cr1—C2	2.078 (15)	C9—H9A	0.9800
Cr2—C23	2.057 (15)	C9—H9B	0.9800
Cr2—C24	2.059 (15)	C9—H9C	0.9800
Cr2—C15	2.074 (16)	C16—C17	1.511 (19)
Cr2—C13	2.077 (13)	C16—H16A	0.9900
Cr2—C14	2.080 (16)	C16—H16B	0.9900
Cr2—C22	2.081 (15)	C17—C18	1.48 (2)
N1—C1	1.163 (16)	C17—H17	1.0000
N2—C2	1.146 (16)	C18—H18A	0.9800
N3—C3	1.168 (17)	C18—H18B	0.9800
N4—C4	1.45 (2)	C18—H18C	0.9800
N4—H4X	0.8900	C19—C20	1.50 (3)
N4—H4Y	0.8900	C19—H19A	0.9900
N5—C5	1.51 (2)	C19—H19B	0.9900
N5—H5X	0.8900	C20—C21	1.54 (3)
N5—H5Y	0.8900	C20—H20	1.0000
N6—C7	1.476 (18)	C21—H21C	0.9800
N6—H6X	0.8900	C21—H21B	0.9800
N6—H6Y	0.8900	C21—H21A	0.9800
N7—C8	1.478 (17)	C25—C26	1.54 (3)
N7—H7X	0.8900	C25—H25A	0.9900
N7—H7Y	0.8900	C25—H25B	0.9900
N8—C10	1.182 (17)	C26—C27	1.45 (2)
N9—C11	1.171 (18)	C26—H26	1.0000
N10—C12	1.148 (16)	C27—H27A	0.9800
N11—C13	1.136 (16)	C27—H27B	0.9800
N12—C14	1.145 (16)	C27—H27C	0.9800
N13—C15	1.135 (17)	C28—C29	1.46 (2)
N14—C16	1.429 (17)	C28—H28A	0.9900
N14—H14X	0.8900	C28—H28B	0.9900
N14—H14Y	0.8900	C29—C30	1.51 (2)
N15—C17	1.496 (17)	C29—H29	1.0000
N15—H15X	0.8900	C30—H30A	0.9800
N15—H15Y	0.8900	C30—H30B	0.9800
N16—C19	1.40 (2)	C30—H30C	0.9800
N16—H16X	0.8900	O1W—H1WA	0.85 (3)
N16—H16Y	0.8900	O1W—H1WB	0.84 (3)
N17—C20	1.491 (18)	O2W—H2WA	0.85 (3)
N17—H17X	0.8900	O2W—H2WB	0.85 (3)
N17—H17Y	0.8900	O3W—H3WA	0.85 (3)
N18—C22	1.143 (17)	O3W—H3WB	0.85 (3)
N19—C23	1.140 (15)	O4W—H4WA	0.84 (3)
N20—C24	1.172 (15)	O4W—H4WB	0.84 (3)
N21—C25	1.49 (2)	O5WA—O5WB	0.80 (16)
N21—H21X	0.8900		
			
N7—Cu1—N5	97.2 (5)	N1—C1—Cr1	176.1 (10)
N7—Cu1—N4	177.7 (6)	N2—C2—Cr1	175.7 (12)
N5—Cu1—N4	83.8 (5)	N3—C3—Cr1	175.7 (12)
N7—Cu1—N6	84.1 (5)	N4—C4—C5	111.8 (17)
N5—Cu1—N6	169.5 (6)	N4—C4—H4A	109.2
N4—Cu1—N6	95.3 (5)	C5—C4—H4A	109.2
N17—Cu2—N15	173.3 (5)	N4—C4—H4B	109.2
N17—Cu2—N16	83.4 (6)	C5—C4—H4B	109.2
N15—Cu2—N16	96.2 (5)	H4A—C4—H4B	107.9
N17—Cu2—N14	96.1 (5)	C4—C5—C6	112 (2)
N15—Cu2—N14	83.9 (4)	C4—C5—N5	106.4 (16)
N16—Cu2—N14	175.7 (6)	C6—C5—N5	107.9 (19)
N21—Cu3—N24	177.3 (6)	C4—C5—H5	110.3
N21—Cu3—N23	94.6 (6)	C6—C5—H5	110.3
N24—Cu3—N23	83.7 (6)	N5—C5—H5	110.3
N21—Cu3—N22	85.1 (6)	C5—C6—H6A	109.5
N24—Cu3—N22	96.6 (5)	C5—C6—H6B	109.5
N23—Cu3—N22	179.4 (6)	H6A—C6—H6B	109.5
N21—Cu3—N1	93.4 (4)	C5—C6—H6C	109.5
N24—Cu3—N1	88.6 (4)	H6A—C6—H6C	109.5
N23—Cu3—N1	88.1 (4)	H6B—C6—H6C	109.5
N22—Cu3—N1	91.4 (4)	N6—C7—C8	109.1 (12)
C12—Cr1—C10	179.4 (6)	N6—C7—H7A	109.9
C12—Cr1—C1	88.6 (5)	C8—C7—H7A	109.9
C10—Cr1—C1	91.9 (5)	N6—C7—H7B	109.9
C12—Cr1—C3	92.0 (5)	C8—C7—H7B	109.9
C10—Cr1—C3	87.6 (6)	H7A—C7—H7B	108.3
C1—Cr1—C3	89.2 (5)	N7—C8—C9	113.6 (12)
C12—Cr1—C11	89.6 (5)	N7—C8—C7	105.4 (11)
C10—Cr1—C11	89.9 (5)	C9—C8—C7	112.3 (14)
C1—Cr1—C11	177.5 (5)	N7—C8—H8	108.5
C3—Cr1—C11	92.6 (5)	C9—C8—H8	108.5
C12—Cr1—C2	88.6 (5)	C7—C8—H8	108.5
C10—Cr1—C2	91.8 (5)	C8—C9—H9A	109.5
C1—Cr1—C2	87.3 (5)	C8—C9—H9B	109.5
C3—Cr1—C2	176.4 (5)	H9A—C9—H9B	109.5
C11—Cr1—C2	90.9 (5)	C8—C9—H9C	109.5
C23—Cr2—C24	177.0 (5)	H9A—C9—H9C	109.5
C23—Cr2—C15	88.6 (5)	H9B—C9—H9C	109.5
C24—Cr2—C15	94.4 (5)	N8—C10—Cr1	178.5 (13)
C23—Cr2—C13	92.9 (5)	N9—C11—Cr1	178.9 (14)
C24—Cr2—C13	87.2 (5)	N10—C12—Cr1	178.0 (12)
C15—Cr2—C13	86.7 (5)	N11—C13—Cr2	175.9 (13)
C23—Cr2—C14	88.1 (5)	N12—C14—Cr2	179.6 (12)
C24—Cr2—C14	88.9 (5)	N13—C15—Cr2	174.5 (13)
C15—Cr2—C14	174.6 (5)	N14—C16—C17	110.3 (11)
C13—Cr2—C14	89.2 (5)	N14—C16—H16A	109.6
C23—Cr2—C22	88.8 (5)	C17—C16—H16A	109.6
C24—Cr2—C22	91.1 (5)	N14—C16—H16B	109.6
C15—Cr2—C22	92.7 (5)	C17—C16—H16B	109.6
C13—Cr2—C22	178.2 (5)	H16A—C16—H16B	108.1
C14—Cr2—C22	91.5 (5)	C18—C17—N15	112.7 (12)
C1—N1—Cu3	125.3 (10)	C18—C17—C16	116.9 (14)
C4—N4—Cu1	108.7 (9)	N15—C17—C16	104.1 (10)
C4—N4—H4X	109.9	C18—C17—H17	107.6
Cu1—N4—H4X	109.9	N15—C17—H17	107.6
C4—N4—H4Y	110.0	C16—C17—H17	107.6
Cu1—N4—H4Y	109.9	C17—C18—H18A	109.5
H4X—N4—H4Y	108.3	C17—C18—H18B	109.5
C5—N5—Cu1	111.5 (10)	H18A—C18—H18B	109.5
C5—N5—H5X	109.3	C17—C18—H18C	109.5
Cu1—N5—H5X	109.3	H18A—C18—H18C	109.5
C5—N5—H5Y	109.3	H18B—C18—H18C	109.5
Cu1—N5—H5Y	109.3	N16—C19—C20	108.4 (15)
H5X—N5—H5Y	108.0	N16—C19—H19A	110.0
C7—N6—Cu1	108.9 (8)	C20—C19—H19A	110.0
C7—N6—H6X	109.9	N16—C19—H19B	110.0
Cu1—N6—H6X	109.9	C20—C19—H19B	110.0
C7—N6—H6Y	109.9	H19A—C19—H19B	108.4
Cu1—N6—H6Y	109.9	N17—C20—C19	107.6 (14)
H6X—N6—H6Y	108.3	N17—C20—C21	107.8 (15)
C8—N7—Cu1	110.8 (8)	C19—C20—C21	116.5 (17)
C8—N7—H7X	109.5	N17—C20—H20	108.2
Cu1—N7—H7X	109.5	C19—C20—H20	108.2
C8—N7—H7Y	109.5	C21—C20—H20	108.2
Cu1—N7—H7Y	109.5	C20—C21—H21C	109.5
H7X—N7—H7Y	108.1	C20—C21—H21B	109.5
C16—N14—Cu2	108.5 (8)	H21C—C21—H21B	109.5
C16—N14—H14X	110.0	C20—C21—H21A	109.5
Cu2—N14—H14X	110.0	H21C—C21—H21A	109.5
C16—N14—H14Y	110.0	H21B—C21—H21A	109.5
Cu2—N14—H14Y	110.0	N18—C22—Cr2	177.7 (13)
H14X—N14—H14Y	108.4	N19—C23—Cr2	176.2 (12)
C17—N15—Cu2	109.7 (7)	N20—C24—Cr2	175.8 (12)
C17—N15—H15X	109.7	N21—C25—C26	107.4 (13)
Cu2—N15—H15X	109.7	N21—C25—H25A	110.2
C17—N15—H15Y	109.7	C26—C25—H25A	110.2
Cu2—N15—H15Y	109.7	N21—C25—H25B	110.2
H15X—N15—H15Y	108.2	C26—C25—H25B	110.2
C19—N16—Cu2	110.0 (11)	H25A—C25—H25B	108.5
C19—N16—H16X	109.7	N22—C26—C27	116.4 (17)
Cu2—N16—H16X	109.7	N22—C26—C25	108.2 (15)
C19—N16—H16Y	109.7	C27—C26—C25	103.5 (14)
Cu2—N16—H16Y	109.7	N22—C26—H26	109.5
H16X—N16—H16Y	108.2	C27—C26—H26	109.5
C20—N17—Cu2	111.1 (10)	C25—C26—H26	109.5
C20—N17—H17X	109.4	C26—C27—H27A	109.5
Cu2—N17—H17X	109.4	C26—C27—H27B	109.5
C20—N17—H17Y	109.4	H27A—C27—H27B	109.5
Cu2—N17—H17Y	109.4	C26—C27—H27C	109.5
H17X—N17—H17Y	108.0	H27A—C27—H27C	109.5
C25—N21—Cu3	108.7 (11)	H27B—C27—H27C	109.5
C25—N21—H21X	109.9	N23—C28—C29	110.3 (13)
Cu3—N21—H21X	109.9	N23—C28—H28A	109.6
C25—N21—H21Y	110.0	C29—C28—H28A	109.6
Cu3—N21—H21Y	110.0	N23—C28—H28B	109.6
H21X—N21—H21Y	108.4	C29—C28—H28B	109.6
C26—N22—Cu3	110.7 (11)	H28A—C28—H28B	108.1
C26—N22—H22X	109.5	C28—C29—N24	107.1 (13)
Cu3—N22—H22X	109.5	C28—C29—C30	117.7 (16)
C26—N22—H22Y	109.5	N24—C29—C30	112.6 (16)
Cu3—N22—H22Y	109.5	C28—C29—H29	106.2
H22X—N22—H22Y	108.1	N24—C29—H29	106.2
C28—N23—Cu3	109.1 (10)	C30—C29—H29	106.2
C28—N23—H23X	109.9	C29—C30—H30A	109.5
Cu3—N23—H23X	109.9	C29—C30—H30B	109.5
C28—N23—H23Y	109.9	H30A—C30—H30B	109.5
Cu3—N23—H23Y	109.9	C29—C30—H30C	109.5
H23X—N23—H23Y	108.3	H30A—C30—H30C	109.5
C29—N24—Cu3	109.7 (10)	H30B—C30—H30C	109.5
C29—N24—H24X	109.7	H1WA—O1W—H1WB	106 (5)
Cu3—N24—H24X	109.7	H2WA—O2W—H2WB	107 (5)
C29—N24—H24Y	109.7	H3WA—O3W—H3WB	106 (5)
Cu3—N24—H24Y	109.8	H4WA—O4W—H4WB	106 (5)
H24X—N24—H24Y	108.2		
			
Cu1—N4—C4—C5	−40 (2)	Cu2—N16—C19—C20	−41.6 (19)
N4—C4—C5—C6	−71 (2)	Cu2—N17—C20—C19	−31.8 (17)
N4—C4—C5—N5	46 (3)	Cu2—N17—C20—C21	94.5 (15)
Cu1—N5—C5—C4	−31 (2)	N16—C19—C20—N17	48 (2)
Cu1—N5—C5—C6	89.2 (15)	N16—C19—C20—C21	−73 (2)
Cu1—N6—C7—C8	38.0 (14)	Cu3—N21—C25—C26	37.3 (16)
Cu1—N7—C8—C9	163.4 (11)	Cu3—N22—C26—C27	153.0 (12)
Cu1—N7—C8—C7	40.1 (14)	Cu3—N22—C26—C25	37.1 (16)
N6—C7—C8—N7	−50.8 (15)	N21—C25—C26—N22	−49.1 (19)
N6—C7—C8—C9	−174.9 (13)	N21—C25—C26—C27	−173.1 (13)
Cu2—N14—C16—C17	39.5 (14)	Cu3—N23—C28—C29	39.2 (15)
Cu2—N15—C17—C18	168.7 (12)	N23—C28—C29—N24	−49.8 (18)
Cu2—N15—C17—C16	41.0 (13)	N23—C28—C29—C30	−177.9 (14)
N14—C16—C17—C18	−178.1 (13)	Cu3—N24—C29—C28	36.3 (15)
N14—C16—C17—N15	−53.1 (15)	Cu3—N24—C29—C30	167.2 (12)

Symmetry codes: (i) −*x*+1, *y*−1/2, −*z*; (ii) −*x*+2, *y*+1/2, −*z*+1.

### (II) Poly[[hexa-µ-cyanido-κ12C:N-hexacyanido-κ6C-hexakis[(R)-propane-1,2-diamine-κ2N,N']dichromiun(III)tricopper(II)] pentahydrate] Hydrogen-bond geometry (Å, º)

**Table:**

*D*—H···*A*	*D*—H	H···*A*	*D*···*A*	*D*—H···*A*
N4—H4*X*···N19^iii^	0.89	2.27	3.153 (16)	171
N5—H5*X*···O5*WA*^i^	0.89	2.24	3.04 (4)	150
N5—H5*X*···O5*WB*^i^	0.89	2.22	2.89 (3)	133
N5—H5*Y*···O4*W*	0.89	2.50	3.177 (17)	133
N6—H6*X*···N18^iii^	0.89	2.56	3.373 (17)	152
N7—H7*X*···N1^i^	0.89	2.49	3.219 (15)	139
N14—H14*Y*···N9^iv^	0.89	2.62	3.360 (16)	142
N15—H15*Y*···N11^ii^	0.89	2.27	3.156 (15)	177
N16—H16*Y*···O1*W*^v^	0.89	2.32	3.159 (16)	158
N17—H17*X*···O2*W*	0.89	2.33	3.132 (18)	150
N17—H17*Y*···N13^ii^	0.89	2.69	3.166 (19)	115
N17—H17*Y*···O4*W*^vi^	0.89	2.60	3.361 (18)	145
N21—H21*X*···O5*WA*	0.89	2.16	3.04 (5)	170
N21—H21*X*···O5*WB*	0.89	2.02	2.88 (3)	163
N21—H21*Y*···N12	0.89	2.51	3.376 (17)	164
N22—H22*X*···N18^iii^	0.89	2.43	3.262 (17)	155
N23—H23*X*···N20	0.89	2.68	3.445 (18)	144
N23—H23*Y*···N9^iv^	0.89	2.20	3.086 (17)	172
N24—H24*Y*···O3*W*	0.89	2.09	2.969 (18)	167
O1*W*—H1*WA*···N19^vii^	0.85 (3)	2.14 (5)	2.972 (16)	167 (16)
O1*W*—H1*WB*···N20	0.84 (3)	2.00 (5)	2.822 (15)	164 (14)
O2*W*—H2*WA*···N10^viii^	0.85 (3)	2.09 (10)	2.811 (17)	143 (15)
O2*W*—H2*WB*···O5*WA*	0.85 (3)	1.78 (9)	2.56 (6)	153 (16)
O2*W*—H2*WB*···O5*WB*	0.85 (3)	2.01 (8)	2.85 (7)	169 (20)
O3*W*—H3*WA*···N18^iii^	0.85 (3)	2.27 (14)	2.982 (19)	142 (20)
O3*W*—H3*WB*···O1*W*^ix^	0.85 (3)	1.92 (10)	2.712 (17)	156 (21)
O4*W*—H4*WA*···N10^i^	0.84 (3)	2.53 (18)	3.104 (17)	126 (18)
O4*W*—H4*WB*···N8^x^	0.84 (3)	2.16 (13)	2.883 (16)	145 (19)

Symmetry codes: (i) −*x*+1, *y*−1/2, −*z*; (ii) −*x*+2, *y*+1/2, −*z*+1; (iii) −*x*+2, *y*−1/2, −*z*+1; (iv) −*x*+1, *y*+1/2, −*z*; (v) *x*+1, *y*, *z*; (vi) *x*+1, *y*+1, *z*; (vii) *x*−1, *y*, *z*; (viii) −*x*+2, *y*+1/2, −*z*; (ix) −*x*+1, *y*−1/2, −*z*+1; (x) −*x*, *y*−1/2, −*z*.
